# Red Roses and Gift Chocolates Are Judged More Positively in the U.S. Near Valentine’s Day: Evidence of Naturally Occurring Cultural Priming

**DOI:** 10.3389/fpsyg.2017.00355

**Published:** 2017-03-20

**Authors:** Vivian Zayas, Gayathri Pandey, Joshua Tabak

**Affiliations:** ^1^Department of Psychology, Cornell University, IthacaNY, USA; ^2^Facebook, Inc., Menlo ParkCA, USA

**Keywords:** attitudes, evaluations, symbols, culture, priming

## Abstract

Attitudes are not static, but constructed at the moment of the evaluation, incorporating temporary contextual influences. How do meaningful events that naturally occur within a culture, such as a national holiday, shape evaluative judgments of objects related to the holiday? We focused on evaluations of red roses and gift chocolates, which are everyday objects, but also iconic of Valentine’s Day in the U.S. We reasoned that if cultural events shape evaluations, then roses and chocolates would be evaluated differently near Valentine’s Day. Using a large and diverse U.S. sample, we found that as Valentine’s Day neared, evaluations of roses and chocolates (but not a comparison object) were evaluated more positively. Increases in positivity of roses and chocolates covaried with their increased cultural relevance, as quantified by the volume of web search queries involving these terms. These findings provide a demonstration of *naturally occurring cultural priming* by which the salience of cultural events shape evaluations.

## Introduction

“Rose is a rose is a rose is a rose” (Stein, 1913) might be true if time and place are held constant. But, it’s unclear whether roses, symbols of love in the U.S., would be evaluated similarly across different times of the year when roses’ cultural relevance differs. Roses are not the only objects imbued with associations of love; chocolates are similar. Would roses and chocolates be evaluated differently when their cultural relevance is peaking (e.g., Valentine’s Day) vs. other times (e.g., 2 weeks before Valentine’s Day)?

Valentine’s Day is one of the U.S.’s most popular holidays (Close and Zinkhan, 2006). For some, it is a time to celebrate love (Wolfinbarger, 1990; Clarke et al., 2005) and express gratitude, commitment, and fidelity to romantic partners (Gonzalez and Koestner, 2006). For others, Valentine’s Day season is anything but joyous, as reflected in recent alternatives to February 14th, such as Anti-Valentine’s Day and Singles Awareness Day parties (Close and Zinkhan, 2009).

Whether Valentine’s Day is loved or loathed, roses and chocolates are pervasive cues around the Valentine’s Day season. In the U.S., there may be nothing more iconic of Valentine’s Day than a gift of red roses and a box of chocolates (Belk, 1979). Each year, approximately $1.7 billion are spent on chocolates and $2.1 billion on flowers around Valentine’s Day (Allen, 2015). For flower purchases, Valentine’s Day ranks No. 1 among all the U.S. holidays (National Online Research, Synovate, 2015).

Our main question is: Do evaluations of roses and chocolates change as Valentine’s Day approaches, and are shifts in evaluations related to shifts in the cultural salience of roses and chocolates? This conjecture is plausible given that evaluative judgments are assumed to be constructed at the moment of the evaluation rather than reflecting trait-like attitudes (Schwarz and Bohner, 2001). According to models of concept activation, attitude objects are represented as a network of interconnected units (e.g., Tulving et al., 1982; Fazio et al., 1986). Each time a person brings to mind a particular object, a unique pattern of activation emerges within the network. The particular representation activated at a given time reflects both chronic influences and contextually triggered temporary influences (Higgins, 1996). It is the contemporaneous representation that affects judgments (Wyer and Srull, 1989). In this manner, contextual cues can influence evaluations. Even time may affect how objects are mentally represented (Eagly and Chaiken, 1993). For example, the distance of objects in space and time can affect the features that come to mind, which in turn can affect how the objects themselves are evaluated (Trope and Liberman, 2003; Fujita et al., 2008).

To illustrate, when evaluating roses, a person brings to mind the mental representation of roses. This may include their physical features, related experiences (past, desired, feared; Berridge et al., 2009; Dai et al., 2010), and relevance to current goals (Ferguson and Bargh, 2004). Not all dimensions of this multifactorial representation will be activated to the same extent at different times. If one were just pricked by a rose, thorns may be relatively more accessible. If one just received roses from one’s partner, roses as a symbol of love may be relatively more accessible. Whichever dimensions are most accessible at a given time are those that will exert the greatest influence on evaluations, in combination with chronic influences.

We hypothesized that one source of contextual influence on evaluations are meaningful events that occur naturally in a culture, such as a National Holiday. We refer to this phenomenon as *cultural priming*. This phenomenon is distinct from past work examining how temporary, contextual influence shape evaluative judgments via standard priming techniques in which researchers deliberately expose participants to stimuli that activate conceptual knowledge (Fazio et al., 1986; Ferguson and Bargh, 2004). To our knowledge, no study has examined how shared meaningful events that naturally occur within a culture, such as a National Holiday, shape evaluative judgments. Here, we asked a large and diverse sample from across the U.S. to evaluate images of red roses and gift chocolates between February 3rd and February 14th of 2015. Although red roses and gift chocolates are objects commonly encountered in everyday life, they are particularly relevant during the Valentine’s Day season^1^. We also asked respondents to evaluate an image of an online dating product as a comparison because it is not a symbol of love, but is associated with relationships or the prospect thereof.

Our main prediction was that as Valentine’s Day nears, roses and chocolates are likely to be evaluated more positively, compared to other times of the year. We reasoned that this increased positivity reflects the cultural relevance of roses and chocolates during the Valentine’s Day season. To obtain a quantitative indicator of the cultural salience of roses and chocolates in the U.S. during Valentine’s Day, we used Google Trends. Google Trends provides the volume of web searches of a particular query for a particular time and region (e.g., How frequently people searched for roses on a particular day in the U.S.). We examined the extent to which daily web search queries for “roses” and “chocolates” predict increases in the positivity of roses and chocolates as Valentine’s Day neared.

## Materials and Methods

### Time Period of Data Collection

Respondents were recruited using Google Consumer Surveys (GCS). We recruited respondents in two waves of data collection. Wave 1 began on 2/3/15. Wave 2 began on 2/12/15. For both waves, the desired *N* was specified at the start of data collection and GCS stopped data collection when the specified *N* was achieved. The rate at which GCS collects data depends on a number of factors not disclosed to the researcher. Because we had no *a priori* predictions regarding how evaluations change *following* Valentine’s Day, we focused on data collected during the time period from 2/3/15 to 2/14/15.

### Sample Characteristics

Our sample consisted of 14,793 respondents. We collected information about respondents’ relationship status and divorce history and other demographic information (e.g., gender, age) was inferred by GCS. However, inferred demographic data are not available for all respondents. Detailed information about the sample composition is outlined in **Table 1**. This study was carried out in accordance with the recommendations of Cornell University’s Institutional Review Board. The committee granted the project Exemption from IRB review because it involved survey procedures, no identifying information was requested, and the content of all measures fell under minimal risk.

**Table 1:** Demographic characteristics of respondents (gender, age, relationship status, divorce status, income, urban density, and geographic region).

Demographic characteristic	*Wave 1* *(2/3–2/7)*		*Wave 2* *(2/12–2/14)*	
	*n*	%	*n*	%
*Gender*				
Male	1793	47.4	5603	50.9
Female	1319	34.9	4334	39.3
Unknown	667	17.7	1077	9.8
*Age*				
18–24	439	11.6	1692	15.4
25–34	542	14.3	2163	19.6
35–44	432	11.4	1760	16.0
45–54	511	13.5	1490	13.5
55–64	592	15.7	1447	13.1
65+	357	9.4	874	7.9
Unknown	906	24.0	1588	14.4
*Relationship Status*				
Single; Not interested in dating	574	15.2	1403	12.7
Single; Interested in meeting someone	408	10.8	1574	14.3
Dating only one person	548	14.5	1787	16.2
Dating more than one person	107	2.8	267	2.4
Partnership/Married	2110	55.8	5894	53.5
Other	32	0.8	89	0.8
*Divorce Status*				
No, never been divorced	2602	68.9	7213	65.5
Yes, have been divorced	893	23.6	2337	21.2
Unknown	284	7.5	1464	13.3
*Income*				
$0-$24,999	297	7.9	1142	10.4
$25,000–$49,999	2220	58.7	5030	45.7
$50,000–$74,999	880	23.3	2698	24.5
$75,000–$99,999	225	6.0	889	8.1
$100,000–$149,999	61	1.6	605	5.5
$150,000+	33	0.9	299	2.7
Prefer not to disclose	33	0.9	292	2.7
Unknown	30	0.8	59	0.5
*Urban Density*				
Urban	1299	34.4	3520	32.0
Suburban	1801	47.7	5612	51.0
Rural	586	15.5	1739	15.8
Unknown	93	2.5	143	1.3
*Geographic Region*				
Midwest	1141	30.2	2927	26.6
Northeast	603	16.0	1806	16.4
South	1087	28.8	3449	31.3
West	935	24.7	2740	24.9
Unknown	13	0.3	92	0.8

*Gender, age, income, urban density, and geographic region were inferred from Google Consumer Survey data. Relationship status and divorce history were self-reported.*

### Statistical Power

We did not have an estimate of the effect size of the Valentine’s Day season on evaluations of roses and chocolates. Because we were relying on increased concept accessibility as it naturally occurs in the U.S. during the Valentine’s Day season, we expected the effect of Valentine’s Day season on evaluations of roses and chocolates to be small ($\eta_{p}^{2}$ > 0.01, equivalent to Cohen’s *d >* 0.2 and *r* > 0.1). Additionally, although the diverse nature of our sample’s characteristics as well as the diverse settings in which they completed the survey (e.g., day, time, place), increase the generalizability of our conclusions, it also requires highly precise estimates to detect the effect given these other extraneous factors. We conducted a *post hoc* power analysis using G^∗^Power (3.1) software (Faul et al., 2007). With *N* = 14,793, an alpha of 0.017 (Bonferroni-corrected), and groups set to 8 (2 waves × 4 relationship status groups); statistical power (1-β) of the present study to detect a small effect ($\eta_{p}^{2}$ = 0.01) was 0.99.

### Procedures and Measures

#### Survey

We used Google Consumer Surveys (GCS), a Google Inc. product available at a cost at www.google.com/insights/consumersurveys/home.

Individuals who are using the Internet and would like to access web content that would normally be behind a pay-wall (e.g., a newspaper article for which access would normally require payment of a nominal fee) are prompted to complete a short survey in exchange for free access to the web content (see Supplementary Figure S1). GCS is used to present short surveys with less than 10 questions, and fewer items are encouraged. Our survey consisted of seven questions: Three questions assessed evaluations of the attitude objects, one relationship status question, one question about divorce status, and two items from an established measure of adult attachment. The results of the analysis involving the attachment questions were inconclusive (see Supplementary Materials).

We assumed that roses and gift chocolates are attitude objects that also symbolize love, more so than an online dating product, which is associated with relationships but not necessarily love. In a separate supplemental study, we obtained empirical support for these assumptions. Specifically, we presented a different sample (*N* = 612; supplemental Study S1) of respondents recruited via Amazon’s Mechanical Turk with the same three images, one at a time, and asked “*When you see this image, to what extent does Love come to mind?*” on a scale from 1 (*not at all*) to 7 (*very much*). Respondents also responded to the following open-ended prompt: “*When you see this image, write down all the words, and/or phrases that come to mind*.” We found support that red roses and gift chocolates are more strongly associated with love and Valentine’s Day, compared to the online dating product (see Supplementary Materials).

The order in which the set of three evaluation questions and the set of two attachment questions were presented was counterbalanced across participants. The relationship status question was presented last, except for a subset (*n* = 2859) of respondents in wave 2, for whom it was presented first, to check if presenting the status question earlier in the survey affected respondents’ evaluations. Analysis of wave 2 data showed that order of the relationship status question did not affect responses on any of the three evaluation questions (all *p*s > 0.263) and will not be discussed further. The divorce status question was always asked last.

#### Evaluation Questions

**FIGURE 1:**
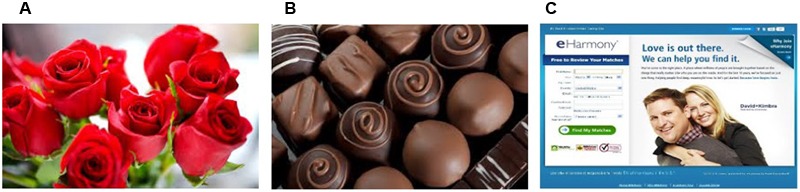
**Images of (A)** roses, **(B)** chocolates, and **(C)** an online dating product used in the survey. For each image, respondents were asked “*Do you like this category?*” on a five-point scale from 1 star (*not at all*) to 5 stars (*extremely*).

Respondents were presented with an image of roses, gift chocolates, and an online dating product (one at a time, in fixed order) and asked “*Do you like this category?*” on a scale from 1 (*not at all*) to 5 (*extremely*) (see **Figure 1** for images).

#### Relationship Status

We asked respondents: “*Which of the following describes your CURRENT relationship status?*” and provided the following options: “*Single; NOT interested in dating*”; “*Single; Interested in meeting someone*”; “*Dating more than one person*”; “*Dating only one person*”; *Partnership/Married*; and “*Other*.” We also asked respondents whether they have ever been divorced (yes, no, or not applicable).

#### Google Trends Search Frequency

Google Trends is a web tool of Google Inc., freely available at www.google.com/trends/ that has been used in past research to quantify changes over time in Internet-based information seeking (Ettredge et al., 2005). We used Google Trends to obtain population-level indicators of the salience of roses, chocolates, and online dating in the U.S. Specifically, we assessed the relative search percentages for “roses,” “chocolates,” and “online dating” in the U.S. between February 3rd to February 16th in 2015 by entering each term individually in the Google Trends tool. Note that the time period in the analyses involving Google Trends differs from the time period used in our primary analyses focused on evaluations. This reflects fundamental differences in the focus of the two analyses. When focusing on evaluations of objects, we limited the analyses to responses collected up to and including Valentine’s Day because we did not have *a priori* predictions about how respondents would evaluate objects *following* Valentine’s Day. In contrast, the analyses involving Google Trends data focus on whether search term frequencies for “roses” and “chocolates” increase near Valentine’s Day and whether such increases covaried with our sample’s mean evaluations. Because the focus of analyses involving Google Trends data are not affected by the same considerations as the primary question, we used all available data to maximize the *N*.

For each Google Trends query, the resulting historical Google Web Search frequency is a relative measure based on the total number of Google searches for the particular term specified in a region during the time period being examined. Google Trends standardizes the maximum query frequency in the time period to 100, and the query frequency at the initial date being examined to zero. Thus, the absolute values of the search frequency are not interpretable; only the relative changes across time within a Google Trends query are interpretable (see Choi and Varian, 2012).

### Data Analytic Approach

#### Coding Temporal Proximity to Valentine’s Week

We coded temporal proximity to Valentine’s Day into one of two groups: *wave 1* refers to responses collected during 2/3–2/7, and *wave 2* refers to responses collected during the days immediately prior to and including Valentine’s Day (2/12–2/14). We used this categorical variable (referred to simply as *wave*) in all analyses. We also tested our hypothesis using a continuous measure of *days until Valentine’s Day* by subtracting the specific date in February from 14 (the yearly date of Valentine’s Day).

#### Statistical tests

We performed three repeated measures ANOVAs to test each pairwise comparison (e.g., roses vs. online dating). Attitude object (e.g., roses vs. online dating product) was the within-person factor and wave (wave 1 vs. wave 2) was the between-person factor. We repeated all analyses to examine whether age or gender moderated the effects. In addition, we repeated all our primary tests to statistically control for respondents’ gender and age by including them in the models as covariates (categorical and continuous, respectively). The results of these secondary analyses were highly similar to those reported here (see Tables S2, S4, S5 in the Supplementary Materials). We chose to report the results without the covariates in the main text, and with covariates in the Supplementary Materials, based on recommendations by Simmons et al. (2011, p. 1363).

We also examined whether gender and age jointly moderated the effect of proximity to Valentine’s Day on evaluations of each attitude object. Specifically, we ran three separate univariate ANOVAs for evaluations of roses, chocolates and online dating, with gender, age, and wave as well as all three-way and two-way interactions as predictors. None of the three-way interactions between gender, age, and wave were statistical significant (see Supplementary Materials).

Additionally, because past research has found that people’s responses to Valentine’s Day vary depending on their relationship status (Morse and Neuberg, 2004; Gonzalez and Koestner, 2006), we tested for its moderating effects. In these analyses, we excluded respondents who selected “other” (*n =* 121) for relationship status. Additionally, we excluded respondents who self-identified as “dating more than one person,” because this group had a considerably smaller sample size (*n* = 374) than the other four groups (*n*s > 1977). This left a sample size of 14,298 for these analyses. We repeated the three repeated measures ANOVAs described above, with relationship status entered as a categorical variable along with its interaction with wave. We performed all pairwise comparisons using Tukey’s honestly significant difference (HSD) test.

#### Adjusting for Multiple Comparisons

Because we tested our full model with three repeated measures ANOVAs (one for each pairwise), we applied a Bonferroni correction and adjusted the criterion for each test to *p* = 0.017.

#### Additional Information

When the homogeneity of variance (HOV) assumption was violated, we report adjusted *df.* To test for significant differences in the magnitude of two dependent correlations (i.e., that share a third variable), we used Steiger’s Z (Z_H_; Hoerger, 2013).

#### Procedural Covariates

We repeated all the analyses described above with experimental factors (e.g., question order, platform, time of day, day of week) as covariates (see Supplementary Materials). The conclusions do not vary when covariates are included in the analyses.

## Results

### Descriptive Statistics

We first assessed descriptive statistics to provide empirical validation of our methodological approach (e.g., by showing that respondent characteristics predict evaluations in expected ways).

#### Evaluations

Evaluation judgments significantly differed across attitude objects [*F*(2,28080) = 14649, *p* < 0.001, $\eta_{p}^{2}$ = 0.50]. Chocolates (*M* = 3.56, *SD* = 1.36) were evaluated more positively than roses (*M* = 3.22, *SD* = 1.35; *p* < 0.001), and roses in turn were evaluated more positively than the online dating product (*M* = 1.67, *SD* = 1.04; *p* < 0.001). Evaluations of roses, chocolates, and the online dating product were all positively correlated with one another. But, notably, the correlation between roses and chocolates (*r* = 0.57, *p* < 0.001) was significantly greater than (*p* < 0.001) the correlation between roses and the online dating product (*r* = 0.25, *p* < 0.001), and also significantly greater than (*p* < 0.001) the correlation between chocolates and the online dating product (*r* = 0.21, *p* < 0.001). This is consistent with our assumption that roses and chocolates are more similar to one another, than the online dating product.

#### Gender

Women, compared to men, expressed stronger positive evaluations of roses [*t*(12289) = 25.55, *p* < 0.001, *d* = 0.45] and chocolates [*t*(12427) = 15.57, *p* < 0.001, *d* = 0.27], but more negative evaluations of the online dating product [*t*(12239) = –2.73, *p* = 0.006, *d* = 0.05].

#### Age

With increasing age, respondents expressed less positive evaluations of roses (*r* = –0.05, *p* < 0.001), chocolates (*r* = –0.12, *p* < 0.001), and the online dating site product (*r* = –0.05, *p* < 0.001).

#### Relationship Status

**FIGURE 2:**
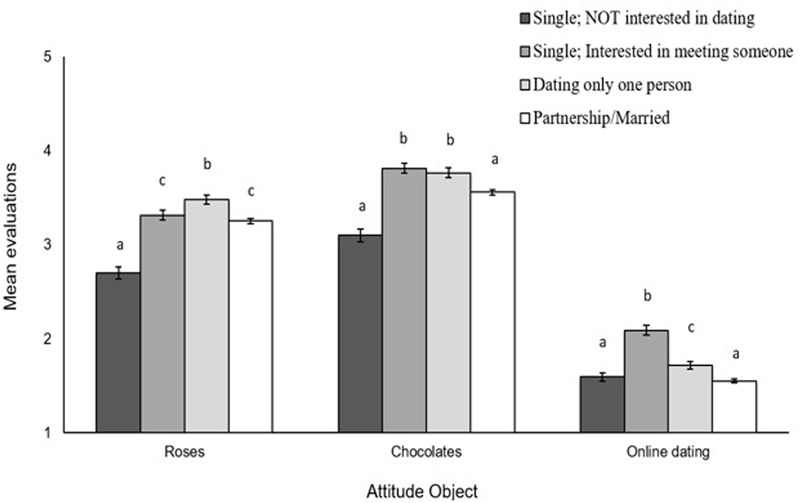
**Mean evaluations of roses, chocolates, and the online dating product as a function of relationship status (single and not interested in dating; single and interested in meeting someone; dating only one person; partnership/married).** Evaluations were made on a scale from 1 (*not at all*) to 5 (*extremely*). Higher numbers reflect greater positivity. Error bars represent 95% CI. Within each object, means with different letters differ significantly at *p* < 0.001 using Tukey’s HSD test.

There was an overall main effect of relationship status on evaluations of roses [*F*(3,14294) = 135.94, *p* < 0.001, $\eta_{p}^{2}$ = 0.028], chocolates [*F*(3,14294) = 117.83, *p* < 0.001, $\eta_{p}^{2}$ = 0.024], and the online dating product [*F*(3,14294) = 154.69, *p* < 0.001, $\eta_{p}^{2}$ = 0.031]. As shown in **Figure 2**, there were two striking differences in evaluations as a function of respondents’ relationship status. First, with regards to evaluations of roses and chocolates, respondents who self-identified as “single and not interested in dating” expressed the least positive evaluations, compared to each of the other three group (*p*s < 0.001). Second, with regard to evaluations of the online dating product, respondents who self-identified as “single and interested in dating” expressed the most positive evaluations, compared to each of the other three groups (*p*s < 0.001).

### Do Evaluations of Roses and Chocolates Change as Valentine’s Day Approaches?

We first computed the zero-order correlations between *days until Valentine’s Day* (*range*: –11 to 0 days, inclusive) and evaluations of the three attitude objects. As Valentine’s Day neared, respondents expressed more positive evaluations of roses (*r* = 0.12, *p* < 0.001; 95%CI = [0.10,0.14]) and chocolates (*r* = 0.11, *p* < 0.001; 95%CI = [0.09,0.12]). Evaluations of the online dating product also became more positive (*r* = 0.034, *p* < 0.001; 95% CI = [0.02,0.05]). But, importantly, *days until Valentine’s Day* correlated more strongly with evaluations of roses than with evaluations of the online dating product (*Z*_H_= 8.46, *p* < 0.001). Similarly, *days until Valentine’s Day* correlated more strongly with evaluations of chocolates than with evaluations of the online dating product (*Z*_H_= 7.01, *p* < 0.001). The correlation between *days until Valentine’s Day* and evaluations of roses did not differ from the correlation involving evaluation of chocolates (*Z*_H_= 1.72, *p* = 0.085).

**FIGURE 3:**
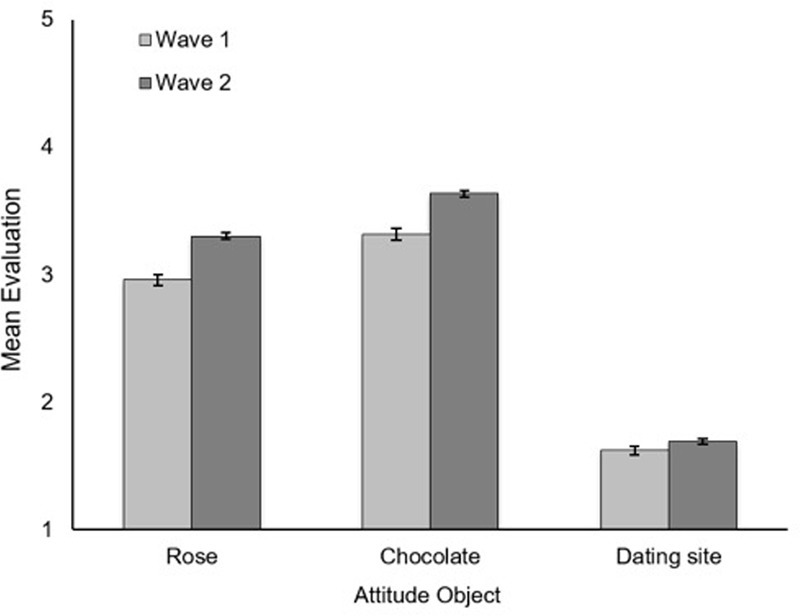
**Bars represent mean evaluations of attitude objects (roses, chocolates, and online dating product) as a function of temporal proximity to Valentine’s Day (wave 1 [2/3–2/7] vs. wave 2 [2/12–2/14]).** Evaluations were made on a scale from 1 (*not at all*) to 5 (*extremely*). Higher numbers reflect greater positivity. Error bars represent 95% CI.

To provide a strong empirical test, we compared temporal changes in evaluations of roses, and of chocolates, to temporal changes in evaluations of the online dating product. As shown in **Figure 3**, compared to the online dating product, the time around Valentine’s Day (wave 2 vs. wave 1) led to more positive evaluation of roses [*F*(1,14791) = 98.50, *p* < 0.001, $\eta_{p}^{2}$ = 0.007]. Similarly, compared to the online dating product, proximity to Valentine’s Day led to more positive evaluations of chocolates [*F*(1,14783) = 75.25, *p* < 0.001, $\eta_{p}^{2}$ = 0.005]. There was no significant difference in the effect of Valentine’s Day on evaluations between roses and chocolates [*F*(1,14783) = 1.35, *p* = 0.25].

In 2015, Valentine’s Day landed on a Saturday. In our data, evaluations varied meaningfully by the day of the week, with more positive evaluations as the weekend approached compared to earlier in the week (See Supplementary Table S1). Appropriately controlling for the effect of day of the week was crucial. Our analyses already statistically control for day of the week (see Data Analytic Approach”). However, to further confirm that our results were not due to day of week, we took the data for Thursday, Friday, and Saturday specific day, we observed heightened positivity of roses and chocolates as Valentine’s Day neared (*p*s < 0.007), but not for the online dating product (*p*s > 0.121). For example, roses were evaluated more positively Friday, February 13th, the day immediately before Valentine’s Day (wave 2) compared to the Friday, February 6th, 1 week earlier (wave 1).

#### Tests of Moderation by Gender, Age, and Relationship Status

Interestingly, neither respondent gender nor relationship status moderated the effect of proximity to Valentine’s Day on evaluations of roses and chocolates (see Tables S2–S4 in Supplementary Materials for descriptives and summary of tests of interactions). Similarly, despite the large age range (18–65+ years old) in our sample, all age groups showed the predicted increase in positivity of roses and chocolates in wave 2 vs. wave 1, with the sole exception being respondents’ ages 18–24 years old who did not show an appreciable increase in positivity of roses. This moderation by age for evaluation of roses [*F*(1,12295) = 11.44, *p* < 0.001, $\eta_{p}^{2}$ = 0.001] was not predicted.

### Do Google Search Term Frequencies for “Roses” and “Chocolates” Predict Increases in Evaluations of Roses and Chocolates in the U.S. as Valentine’s Day Approaches?

**FIGURE 4:**
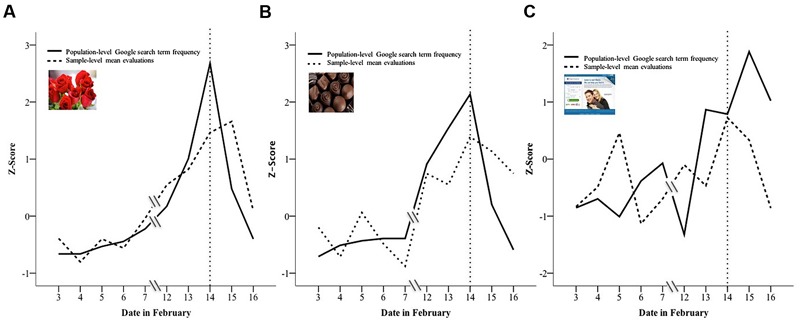
**Sample-level mean evaluations (dotted line) and population-level Google search term frequency (solid line) as a function of date in February for roses (A)**, chocolates **(B)**, and an online dating category **(C)**. Sample-level mean evaluations and Google search term frequency are reported in standardized units to facilitate comparison. The dotted vertical line on 14th of February on the *x*-axis indicates Valentine’s Day. To provide a better estimate of the relationship between search terms and evaluation of objects, we used evaluation data from all available days (2/3/15 to 2/16/15). Google search term frequency is a relative measure based on the total number of Google searches for the particular term specified in the U.S. during the time period being examined. The maximum query frequency in the time period specified is standardized to 100, and the query frequency at the initial date being examined is standardized to be zero by Google Trends. Thus, the absolute values of the search frequency are not interpretable; only the relative changes across time within a Google Trends query are interpretable.

An examination of the results obtained from Google Trends data supports our intuitions that the salience of roses and chocolates increases in relevance near Valentine’s Day. As shown in **Figure 4**, the frequency of searching for “roses” (**Figure 4A**) and “chocolates” (**Figure 4B**) increased as Valentine’s Day approached, peaked on Valentine’s Day, and decreased following Valentine’s Day. The volume of search queries for “online dating” (**Figure 4C**) did not show the same clear pattern. In fact, in 2015, the frequency of searching for “online dating” peaked the day *after* Valentine’s Day.

Do these trends in U.S. web search activity in the time period between February 3rd and February 14th of 2015 reflect cyclical, seasonal patterns that occur annually in the U.S. around Valentine’s Day, or are these trends unique to 2015? To address this question, we conducted auxiliary analyses using all available data from Google Trends (2004–2016; see Supplementary Materials). In every single year between 2004 and 2016, we found a prominent peak in search frequencies for “roses” and “chocolates.” Moreover, the volume of search queries for “online dating” did not show the same cyclical seasonal pattern. This lack of seasonal web activity for “online dating” further supports the assumption that online dating is not necessarily associated with love and Valentine’s Day. Moreover, the trends data for “online dating” also offers a comparison, showing that it is not the case that people are simply conducting more searches around Valentine’s Day or conducting more searches related to relationships.

Importantly, we predicted that the increased positivity in evaluations observed in our sample should covary with population indicators of web search frequencies for “roses” and “chocolates.” Indeed, the search frequency of “roses” and “chocolates” on a given day predicted their respective mean evaluations in our sample on that day. To quantify this association, we used day (e.g., February 3rd) as the unit of analysis. The zero-order correlations between sample-level mean evaluations and population-level Google search term frequency were statistically significant for roses [*r*(10) = 0.81, SE = 0.38, *p* = 0.004; 95%CI = [0.36,0.95]], and chocolates [*r*(10) = 0.68, SE = 0.38, *p* = 0.03; 95%CI = [0.09,0.92]], but not for the online dating product [*r*(10) = 0.20, SE = 0.38, *ns*; 95%CI = [–0.49,0.74]]. These patterns provide evidence that not only do evaluations of roses and chocolates become more positive as Valentine’s Day approaches, but that their rate of increase in positivity maps on to the rate of increase in people’s searching patterns for these objects (which also increases as Valentine’s Day approaches).

## Discussion

### Demonstration of Naturally Occurring Cultural Priming

When making evaluative judgments, people do not simply bring to mind a static attitude from memory. Instead, judgments are constructed *at the moment*, shaped by a variety of chronic and temporary influences (Schwarz and Bohner, 2001). The present work shows that culturally meaningful events, such as Valentine’s Day in the U.S., can alter evaluative judgments, providing the first demonstration of *naturally occurring cultural priming*. Using a large and diverse U.S. sample, we show that instead of being evaluated similarly across time, evaluations of roses and chocolates increase in positivity as Valentine’s Day approaches. As evidence that this increased positivity reflects the cultural salience of Valentine’s Day, we found that web search frequencies for “roses” and “chocolates” also increased as Valentine’s Day approached, and that increases in web search frequencies significantly predicted our sample’s mean evaluative judgments of roses and chocolates.

A key feature of the present work is that we did not explicitly (or even implicitly) remind respondents of Valentine’s Day; instead, respondents simply evaluated images of roses and chocolates, but did so at a time when Valentine’s Day was near or far. Thus, this is a demonstration of naturally occurring cultural priming, which is starkly different from experimentally manipulated standard priming methods used to activate conceptual knowledge (e.g., unscrambling letters to form the word “Valentine’s Day”). These findings add to the rich literature on the intersection of culture and social cognition. Whereas past work has focused primarily on differences between cultures (East Asians vs. northern Europeans; Oyserman and Lee, 2008) or between groups within a culture (high vs. low in cultural assimilation; Hong et al., 2000), here we demonstrate that concept activation can vary within a population as a result of changes in cultural salience.

### Culturally Shared vs. Idiosyncratic Associations

It is noteworthy that overall evaluative judgments were related to demographic characteristics in predictable ways: for example, as one might expect, women judged roses and chocolates more favorably than men, and singles interested in meeting someone judged the online dating product more favorably than singles uninterested in dating. These results are important because they validate our methodological approach and increase our confidence that respondents’ reports reflect their evaluative judgments.

Importantly, however, none of the demographic characteristics moderated the effect of proximity to Valentine’s Day on evaluations of roses and chocolates. That is, the increased positivity of roses and chocolates closer to Valentine’s Day occurred for both men and women, for individuals of all relationship statuses, and for all age groups (except for 18–24 year olds’ evaluations of roses). Null findings are typically difficult to interpret. However, given the high statistical power (>0.99) of our study to detect even a small effect, the absence of differences is informative.

Indeed, the finding that individuals regardless of their relationship statuses and gender showed similar increases in positivity of roses and chocolates as Valentine’s Day approached is noteworthy. The meaning of Valentine’s Day depends on one’s personal, social, and relational goals. To the extent that Valentine’s Day serves as a celebration and validation of one’s relationship, it should activate positive thoughts particularly for those in a relationship (Close and Zinkhan, 2006; Gonzalez and Koestner, 2006). But, the holiday’s increased focus on relationship status (e.g., Wood and Wilson, 2003) and romantic relationships (Hoerger et al., 2012; Chopik et al., 2014) may make those who are single, whether by choice or circumstance, to feel alienated or pressured to find a date or mate (Morse and Neuberg, 2004), and those who are involved in fledgling, noncommittal, or otherwise less stable relationships to experience heightened relational worries and breakups (Morse and Neuberg, 2004). But, in our study, we found no evidence that this was the case.

The absence of significant moderation as a function of gender and relationship status suggests that what is being activated in memory are culturally shared associations of *roses*—*love and chocolates*—*love*, not associations that reflect idiosyncratic experiences (Karpinski and Hilton, 2001; Olson and Fazio, 2004). Indeed, in the U.S., associations with Valentine’s Day are likely to form early; a common ritual involves children making and exchanging Valentines with classmates, friends, and family. Moreover, given that the present study made no explicit mention of Valentine’s Day, this may have decreased any reactance (negative or positive) that may be triggered by explicit reminders of Valentine’s Day.

### A Conceptual Priming Explanation

What are the mechanisms by which cultural events, such as a national holiday, shape evaluative judgments? From a conceptual priming framework (Tulving et al., 1982), the effect of cultural events on information processing depends on the contextual meaning surrounding the cultural event. In the case of Valentine’s Day in the U.S., the cultural context involves love. As the salience of Valentine’s Day and the relevance of associated symbols increase, so too should the salience of the *roses—love* and *chocolates—love* association. That is, as Valentine’s Day nears, roses and chocolates should be evaluated based on their associations with love, more so than at other times of the year. And because the concept of love is positive (Hermans et al., 2000), this should lead to increased positivity of roses and chocolates as Valentine’s Day approaches.

The entirety of the results, including the supplemental study and historical analyses of all existing Google Trends data, support this account. First, the supplemental study shows that roses and chocolates are symbols of love. In the language of conceptual priming, people possess *roses—love* and *chocolates—love* associations. Second, our analyses of historical Google Trends data provide quantitative indicators of the salience of roses and chocolates near Valentine’s Day. In every single year with available Google Trends data (2004–2016), web searches for “roses” and “chocolates” show demonstrable peaks during the Valentine’s Day season. This was not the case for “online dating.” Moreover, further supporting the idea that people possess *roses*—*love* and *chocolates—love* associations, there are seasonal peaks of web searches for the term “love” too (see Supplementary Materials). Finally, our supplemental study shows that objects more strongly associated with the concept love, are in fact evaluated more positively. Taken together, a conceptual priming account provides the most parsimonious explanation for how cultural events associated with a national holiday affect evaluative judgments.

### Alternative Accounts

We consider three alternative explanations for the observed findings: mere exposure, cognitive dissonance, and goal pursuit. A mere exposure account (Zajonc, 1980) predicts that objects that are encountered more frequently (in this case, roses and chocolates during the Valentine’s Day season) increase in positivity. A meta-analysis of mere exposure studies (Bornstein, 1989), however, suggests that this is unlikely. Mere exposure effects are typically studied using novel or unfamiliar stimuli (e.g., Chinese ideographs) that lack conceptual meaning. Roses and chocolates are anything but novel or lacking in conceptual or affective meaning. Moreover, high exposure can *decrease* liking for positive attitude objects (Bornstein, 1989) via habituation (Dijksterhuis and Smith, 2002) or hedonic adaptation (Frederick and Loewenstein, 1999), and can even elicit ambivalence (Brooks and Highhouse, 2006). Finally, it is worth noting that marketing campaigns for Valentine’s Day begin around January 2nd (Gursky, 2016), and is characterized by shops stocking their shelves with Valentine’s Day gifts. But, our data show a pronounced increase in positivity of roses and chocolates the week prior to Valentine’s Day. Thus, a mere exposure account is inconsistent with the observed findings.

Another explanation is that the increased positivity for roses and chocolates closer to Valentine’s Day reflects differences in the functional relevance of these objects as gifts. Both a cognitive dissonance (Henkel and Mather, 2007) and goals framework (Ferguson and Bargh, 2004) would predict that individuals who typically spend more on roses and chocolates near Valentine’s Day would show greater positivity towards these objects as the holiday neared. From a cognitive dissonance framework, increased positivity of roses and chocolates arise to reduce the dissonance elicited by having purchased these objects at a nontrivial cost (Henkel and Mather, 2007). From the goals literature, increased positivity of roses and chocolates would facilitate purchase behavior. Our data, however, are not consistent with these accounts. Men spend about twice as much as women on Valentine’s Day gifts, and single people spend more than married ones (Chance and Norton, 2010; Wilson et al., 2015); yet, neither gender nor relationship status appreciably moderate the effect of Valentine’s Day on evaluations of roses and chocolates.

### Limitations and Future Directions

The present work provides the first demonstration of changes in evaluations of objects as a function of proximity and salience of cultural events. Because the focus of the present study was on culturally primed variation in positivity toward classic symbols of romantic love, we chose to ask respondents to evaluate images of red roses and boxed chocolates – iconic symbols of love in the U.S. We included an image of an online dating product as a comparison, because it is associated with romantic relationships and their prospect, but not necessarily with love. But, it is important to consider that the image of an online dating product may have activated other associations that are not relevant to relationships or associations unique to seeking partners through online dating sites. Indeed, evaluations of the online dating product were overall negative, although respondents who were single and interested in meeting someone showed more favorable judgments. Additionally, we used an image for a specific online dating product, and this may have activated associations that are particular to the specific site, rather than relationships or online dating in general. Although this concern cannot account for the increased positivity in evaluations of roses and chocolates as Valentine’s Day approaches, future research might examine whether evaluations of other images associated with relationships increase as Valentine’s Day approaches.

One important direction for future research is to examine the extent to which other holidays or events within a culture give rise to similar naturally occurring priming effects on attitudes. For example, does corn increase in positivity as Thanksgiving approaches? Likewise, are pumpkins evaluated more favorably as Halloween nears? Do bald eagles lead to stronger associations with freedom during Independence Day? Moreover, do some cultural events lead to increased negativity, e.g., commemorating 9/11 attacks on the World Trade Center?

Additionally, these findings raise interesting questions for future inquiry regarding the effects of Valentine’s Day in the U.S.: Would roses be seen as more beautiful, and chocolates as more delicious, on Valentine’s Day compared to another day? Would these effects manifest themselves not only in people’s judgments of love symbols but in their experience and consumption of these objects as well? Another important avenue for future work is exploring the mechanisms by which cultural events (1) increase the activation of culturally relevant concepts in the minds of members living within a given culture, and (2) how they lead to increases in positivity.

## Conclusion

Our study shows that cultural events provide a backdrop that shape evaluative judgments—the first demonstration of *naturally occurring cultural priming*. Contrary to the Stein’s famous quote, rose is *not* a rose is *not* a rose is *not* a rose.

## Author Contributions

VZ and JT came up with the hypotheses, study design, and materials for the main study. VZ and GP designed the study and developed the materials for the supplemental study. VZ analyzed the data for the main study, and VZ and GP analyzed the data for the supplemental study. GP generated the historical analysis of the Google Trends Data. VZ, GP, and JT wrote the ms.

## Conflict of Interest Statement

The authors declare that the research was conducted in the absence of any commercial or financial relationships that could be construed as a potential conflict of interest.

## Footnotes

1 A separate study (see Supplemental Study S1) showed that red roses and chocolates are associated with love more than an online dating product, and that the strength of an object’s association with love predicts the extent to which the object is judged positively.
