# Supplementary material for: Red Roses and Gift Chocolates Are Judged More Positively in the U.S. Near Valentine’s Day: Evidence of Naturally Occurring Cultural Priming
Source: Front Psychol. 2017 Mar 20;8:355. doi: 10.3389/fpsyg.2017.00355 (PMC5357630; doi:10.3389/fpsyg.2017.00355)
Supplement: Supplementary file 1 [file Supplementary_Materials.pdf]

Supplementary Material

Red roses and gift chocolates increase in positivity in the U.S. near Valentine's Day: Evidence of naturally occurring cultural priming

Vivian Zayas*, Gayathri Pandey, Joshua Tabak

*** Correspondence:** Vivian Zayas: vz29@cornell.edu

1 Supplementary Data

Procedural Variables

Our research included a number of procedural factors, such as the *order* that respondents completed the items in the survey (three evaluation questions first followed by two attachment questions vs. two attachment questions first followed by the three evaluation questions), slight variation in *question wording* presented to respondents ("*Do you like this category?*" vs. "*Do you like this category of product*"?), and the *platform* used to complete the survey (Android vs. desktop). In addition, *day of the week* (Tuesday, Wednesday, Thursday, Friday, or Saturday) and *time of day* are situational factors that could have affected evaluations.

We repeated the main analyses involving proximity to Valentine's Day (wave 1 vs. wave 2) with all the above-mentioned procedural variables entered as covariates. All variables were entered as categorical variables, except for the time of day, which was entered as a continuous variable. A summary of the effects of these variables is provided in Table S1. As shown, the conclusions regarding the effect of proximity to Valentine's Day on evaluation of roses and chocolates remain unchanged when we statistically controlled for these factors.

Analysis of adult romantic attachment

We explored the moderating role of adult attachment style. Specifically, we examined the possibility that attitudes towards love symbols may vary as a function of a person's adult attachment style, and that a respondents' adult attachment style might moderate the effect of Valentine's Day on attitudes towards love symbols.

Adult romantic attachment questions. To assess adult attachment, respondents completed two items from a widely used and well-established measure of adult attachment (ECR-R; Fraley, Waller, & Brennan, 2000). Due to constraints around the total length of the GCS instrument, it was only possible to administer a single item from the attachment anxiety and attachment avoidance scales. Single item measures are useful to minimize disengagement associated with multiple item measures and have been used successfully to assess other important individual differences (Wanous, Reichers, & Hudy, 1997). To assess attachment anxiety, we asked: "*To what extent does the following statement describe you: 'I prefer not to be too close to my romantic partner.'*" To assess attachment avoidance, we asked: "*To what extent does the following statement describe you: 'I worry a lot that my partner will leave me.'*" Both items were answered on a scale from 1 (*not at all*) to 5 (*extremely*). In the present

study, the zero-order correlations involving responses on these two items and evaluations of roses, chocolates, and an online dating product are highly consistent with extant findings (Hazan & Shaver, 1987) and add confidence to the validity of these single-item measures.

Statistical Test. We tested for the moderating effects of relationship status and adult attachment simultaneously, using the same strategy as described in the Method section. Specifically, for these analyses, we excluded respondents who selected the response "other" (.8% of the sample; n =121) for relationship status. Additionally, we excluded respondents who self-identified as "dating more than one person," because this group had considerably smaller sample sizes (n = 375) than the other four groups (ns > 1977). This left a sample size of 14,298 for these analyses. Additionally, for the sake of simplicity, these analyses focus on a composite evaluation score of roses and chocolates. The conclusions drawn here did not vary when we examined evaluations of roses and chocolates separately. We repeated the three repeated measures ANOVAs but additionally entered relationship status as a categorical variable and attachment anxiety and attachment avoidance as continuous predictors, along with all interactions involving these predictors and wave (wave 1 vs. wave 2).

Results. The results of ANOVAs examining the effect of adult attachment anxiety, attachment avoidance, relationship status, and temporal proximity to Valentine's Day are reported in Table S3.

Respondents characterized by higher romantic attachment anxiety expressed more positive evaluations of love objects (zero-order r (14298) = .12, p < .001, 95% CI = [.10, .13]), whereas those characterized by higher romantic avoidant attachment expressed more negative evaluations (zero-order r (14298) = -.06, p < .001, 95% CI = [-.08, -.04]). Both attachment anxiety and attachment avoidance were positively correlated with evaluations of the online dating product (attachment anxiety: (zero-order r (14298) = .21, p < .001, 95% CI = [.19, .22]); attachment avoidance: (zero-order r (14298) = .13, p < .001, 95% CI = [.11, .14])).

Both attachment anxiety and attachment avoidance interacted with Valentine's Day week, reflected by a two-way interaction between each scale and temporal proximity to Valentine's Day (wave 1 vs. wave 2). Each of these two-way interactions, however, occurred in the presence of a three-way interaction with relationship status. To illustrate these results, we plotted the data in Figure S2 (for attachment anxiety) and Figure S3 (for attachment avoidance). In each figure, each panel represents the data for one of the relationship status groups. The y-axis represents the mean evaluation of love symbols (composite score) and the x-axis represents responses on the particular attachment question. Given that we used a single item to assess attachment anxiety and avoidance, for the sake of representing all the data, we treated responses to each question as nominal levels ranging from 1 (*not at all*) to 5 (*extremely*).

Testing the combined effects of gender and age on the effect of proximity to Valentine's Day

We also examined whether gender and age jointly moderated the effect of proximity to Valentine's Day on evaluation of attitude objects. We ran three separate univariate ANOVAs for evaluations of roses, chocolates and online dating, and with gender, age, and wave as well as all three-way and two-way interactions as predictors. None of the three-way interactions between gender, age, and wave reached statistical significance.

Attitude object evaluation (e.g., evaluation of roses) was entered as the dependent variable and wave (wave 1 vs. wave 2), gender and age were entered as between-person factors. For roses, we found a main effect for wave ($F(1, 12274)=138.74$, $p<.0001$), such that respondents evaluated roses significantly more positively during wave 2 ($M=3.30$, $SD=1.32$) than during wave 1 ($M=2.97$, $SD=1.37$). As already reported in the main study, we also found main effects for gender ($F(1, 12274)=408.66$, $p<.0001$) and age ($F(5, 12274)=15.505$, $p<.0001$). Overall, women evaluated roses more positively ($M=3.57$, $SD=1.28$) compared to men ($M=2.99$, $SD=1.31$). And, respondents in the age groups of 18-24 and 25-34 evaluated roses more positively compared to other age groups (i.e., 35-44, 45-54, 55-64, and 65+; ps<.0001). However, the three-way interaction between wave, age and gender was not significant ($F(5, 12274)=1.43$, $p=.21$).

For chocolates, similar to roses, we found a main effect for wave ($F(1, 12274)=97.53$, $p<.0001$), such that respondents evaluated chocolates significantly more positively during wave 2 ($M=3.63$, $SD=1.33$) than during wave 1 ($M=3.31$, $SD=1.42$). Again, similar to roses, for chocolates we found main effects for gender ($F(1, 12274)=192.91$, $p<.0001$) and age ($F(5, 12274)=59.08$, $p<.0001$). Overall, women evaluated chocolates more positively ($M=3.79$, $SD=1.30$) compared to men ($M=3.43$, $SD=1.36$). And, respondents in the age group of 18-24 and 25-34 evaluated chocolates more positively compared to other age groups (i.e., 35-44, 45-54, 55-64, and 65+; ps<.0001). However, the three-way interaction between wave, age, and gender was not significant ($F(5, 12274)=2.17$, $p=.054$).

For online dating too, we found a main effect for wave ($F(1, 12274)=11.25$, $p=.001$), such that respondents evaluated online dating significantly more positively during wave 2 ($M=1.68$, $SD=1.04$) than during wave 1 ($M=1.62$, $SD=1.01$). Unlike for roses and chocolates, gender did not have a main effect ($F(1, 12274)=3.56$, $p=.06$). That is, women ($M=1.65$, $SD=1.03$) and men ($M=1.70$, $SD=1.04$) did not differ in their evaluation of online dating. Age on the other hand had a significant main effect ($F(5, 12274)=3.28$, $p=.006$). Specifically, respondents in the age group of 25-34 evaluated online dating more positively ($M=1.78$, $SD=1.08$) compared to respondents in the age group of 55-64 ($M=1.61$, $SD=1.01$; no other group differed from each other, ps>.12). However, again, the three-way interaction between wave, age and gender was not significant ($F(5, 12274)=.190$, $p=.966$).

II Supplemental analyses: Does the salience of “roses,” “chocolates,” and “love” increase as the Valentine’s Day season approaches? Analyses using Google Trends data

Intuitively, one assumes that “love,” and its various associations, are in the air (or on the mind) during the Valentine’s Day season. But, is there empirical evidence of the salience of such concepts in U.S. culture? To quantify the magnitude of the cultural salience of love and love related symbols (i.e., roses and chocolates) around Valentine’s Day in the U.S., we turned to Google Trends™. Google Trends™ provides a measure of the search volume for entered queries per day in a given region (e.g., How frequently people in a region searched for “roses”).

Past work has used Google Trends™ to quantify population level activities that involve web searches. Given the high volume of searches, it is considered a reliable indicator of population level information seeking, for example, as related to medical content (Cooper et al., 2005), sexual content (MacInnis & Hodson, 2015), mental health (Ayers, Althouse, Allem, Rosenquist, & Ford, 2013), and purchases (Choi & Varian, 2012). Starting from the earliest uses, Google Trends™ has shown to be sensitive to trends in populations over time, and been used for forecasting economic indicators, such as U.S. unemployment (Ettredge et al., 2005), and to predict influenza-like diseases (Ginsberg et al., 2009; Polgreen et al., 2008).

In the present analyses, we used all the available data from Google Trends™ (2004 to 2016) to examine whether search frequencies in the U.S. for “love,” “roses,” and “chocolates” show seasonal increases around Valentine’s Day. As a comparison, we also examined the search frequency of “online dating,” which is associated with relationships, but not necessarily considered a symbol of love (see Supplemental Study, pp. 19–38 in the Supplemental Materials).

Method

We performed a series of Google Trends queries to derive the volume of Google web searches involving the terms "*love*," "*roses*," "*chocolates*," and "*online dating*" (each term entered individually in the Google Trends tool) in the U.S. We performed the Google Trends queries in two ways: a) we looked at the search frequency for each of these terms using all existing data (2004 to present), and b) we examined the search frequency for each of these terms only for the months of January and February for each year between 2004 and 2016. The URLs to reproduce the plots of search term frequencies shown in Fig. S4 are as follows: For roses: https://www.google.com/trends/explore#q=roses&geo=US&date=1%2F2004%20150m&cmpt=q&tz=Etc%2FGMT%2B5. For chocolates: https://www.google.com/trends/explore#q=Chocolates&geo=US&date=1%2F2004%20150m&cmpt=q&tz=Etc%2FGMT%2B5. For online dating: https://www.google.com/trends/explore#q=Online%20dating&geo=US&date=1%2F2004%20150m&cmpt=q&tz=Etc%2FGMT%2B5. For love: https://www.google.com/trends/explore#q=Love&geo=US&date=1%2F2004%20150m&cmpt=q&tz=Etc%2FGMT%2B5

Importantly, for each Google Trends™ query, the resulting historical Google Web Search frequency is a *relative* measure based on the total number of Google searches for the particular term (e.g., "roses") specified in a region (e.g., U.S.) during the time period being examined (e.g., 2015). Google Trends standardizes the maximum query frequency in the time period to 100, and the query frequency at the initial date being examined to zero. Thus, the absolute values of the search frequency are not interpretable; only the relative changes across time within a Google Trends query are interpretable.

Results

Prominent yearly seasonal shifts in volume of web search for "roses, "chocolates," and "love." Figure S4 shows the volume of Google web searches for "roses," "chocolates," "love," and "online dating" between 2004 and 2016. What is apparent and most striking is the regularity of seasonal patterns of population-level web search activity for roses (panel A) and chocolates (panel B). Specifically, for every year between 2004 and the present, there are at least two prominent peaks in the search term frequency of "roses": one peak is associated with Valentine's Day and the second peak is associated with Mother's Day. In most years, the highest volume of Google search queries for roses occurs on or near February 14th (Valentine's Day). Likewise, a similarly striking pattern of seasonal activity in Google web searches is observed for chocolates. For every year between 2004 and the present, there are two prominent peaks in the volume of search queries: The highest volume of activity occurs around the end of December, and the next highest volume of activity is associated with Valentine's Day. Importantly, in stark contrast, for online dating (panel C), there was no clear visible seasonal pattern.[1] This indicates that the increased web searches for roses and chocolates as Valentine's Day approaches does not simply reflect an increase in web searches in general (for any term). Finally, for every year between 2004 and the present, search term frequency for love (panel D) increases near

[1] There was a large increase in search term frequency during 2006. Analyses excluding this time frame were highly similar: search queries for online dating do not appear to fluctuate with Valentine's Day.

Valentine's Day, although this seasonal increase is less pronounced than the pattern observed for roses and chocolates.

For the interested reader, we also plotted the search frequencies for only the months of January and February. By doing so, we zoomed in on the data and can see the approximate onset of the increased volume in web searches during the Valentine's Day season. In every year between 2004 to 2016 (see Figures S8 to S20 in the Appendix), there is a clear increase in the search frequency of roses, chocolates and love (but not of online dating) starting approximately the week before Valentine's Day.

As a final point, because the search frequencies within a Google Trends query are standardized from 0 (min) to 100 (max), only relative changes across time for a given Google Trends query are interpretable (see Method section). Thus, it is not possible to compare differences across queries that involve different terms (e.g., comparing volume of searches for "roses" in panel A to searches for "chocolates" in panel B in Figure S4). For this we reason, we also computed the volume of search frequency when all four terms were entered simultaneously in a Google Trends query. This allows for a more direct comparison of differences in the volume across the different entries. These results are plotted in Figures S21 to S24 in the Appendix. Overall, the volume of searches involving "love" are orders of magnitude greater than searches involving "roses," "chocolates," and "online dating."

Conclusion

Overall, analyses of Google Trends™ data support our premise that there are yearly seasonal changes around Valentine's Day in the salience of roses and chocolates, as quantified via the web search frequency of these terms. Moreover, consistent with the idea that love concepts are heightened during the Valentine's Day season, web search frequency for love is also high around Valentine's Day. Importantly, it isn't the case that people are simply conducting more web searches around Valentine's Day or conducting more web searches related to relationships. The volume of web search queries for online dating, which is related to relationships but not necessarily love, did not show the same cyclical seasonal increase in salience.

III Supplemental Study 1: How does heightened accessibility of the concept *love* relate to evaluation of attitude objects?

The primary goal of the main study was to investigate the hypothesis that as Valentine's Day approaches roses and chocolates increase in positivity, reflecting their increased cultural relevance as symbols of love. This prediction is grounded in the basic cognitive principle of accessibility. Specifically, we reasoned that because the concept of love is highly positive (Kent, 2010), as an object's association with love becomes more accessible, the object will be evaluated more through its association with love, leading to stronger positive evaluative judgments of the object.

Extant research has demonstrated that increased accessibility of the concept love affects judgments about relationships and relationship partners, as well as information processing more broadly (Wlododarski & Dunbar, 2014). But, the extent to which increased accessibility of the concept love affects evaluative judgments of generic symbols of love, such as an image of red roses and a box of gift chocolates as used in the main study, has not been established. Given that this is a crucial assumption in our reasoning, we conducted a supplemental study.

The goal of the present supplemental study was twofold: First, we aimed to examine whether an image of red roses and a box of gift chocolates are more likely associated with the concept of love, compared to an image of an online dating product, which we used for comparison because it is associated with relationships but not necessarily viewed as a symbol of love. Second, we tested the hypothesis that the greater an object's association with the concept of love, the more positively the object will be judged. In other words, objects for which the concept of love is spontaneously activated upon viewing will be judged more favorably, compared to objects for which the concept of love is weakly, or not at all, activated.

Method

Participants

We recruited 612 respondents (*Mage* = 35.03 years, *SD* = 11.42 years) from all over the US from diverse demographic groups using Amazon's Mechanical Turk (see Table S6 for demographic information). Of these, 12 respondents had missing responses to two of the Likert accessibility measures (9 for chocolates, 3 for online dating product), leaving a sample of 600 for analyses involving the Likert accessibility measure.

Procedures and measures

Survey. Similar to the main study, respondents were presented with the same three images (roses, chocolates, and an online dating product) and asked to provide their evaluations of each object. In addition, to assess the extent to which respondents associated each object with love, respondents were presented with the same three images, one at a time, and asked "*When you see this image, to what extent does Love come to mind?*" on a Likert scale from 1 (*not at all*) to 7 (*very much*). Respondents also completed an open-ended question about each object. Specifically, for each image they were presented with the following prompt: "*When you see this image, write down all the words and/or phrases that come to mind*." They were given 30 seconds to write about each image. Finally, we collected information about respondents' relationship status, divorce history, and basic demographics.

Procedural variables. Respondents were randomly assigned to complete the open-ended accessibility questions first followed by the evaluation questions or the evaluation questions first

followed by the open-ended accessibility questions. The Likert accessibility questions were always answered last.

Coding open-ended responses. For the open-ended accessibility question, we analyzed the first descriptor listed by the respondents, given that listing order reflects associative strength (Fazio, Williams, & Powell, 2000) with items mentioned first presumably more accessible than those listed later (Bargh, Lombardi, & Higgins, 1988; Higgins, King, & Mavin, 1982). A research assistant who was blind to the hypothesis coded the content of the first descriptor generated by each respondent. Our main focus in the coding process was to identify if the first descriptor referred to Valentine's Day or love, or relationships. However, descriptors that did not fall into these categories were coded into the following 6 categories: Objective features, gifts, commercialism, other descriptor with positive connotations, other descriptor with negative connotations, or other descriptor with no evaluation implied. Each descriptor was classified into one and only one category. Examples of respondents' actual responses for each category across the three attitude objects are shown in Table S7.

Next, for each object, we computed 8 variables, one for each of the eight categories, and coded whether the first response belonged to the particular category (coded 1, if yes, and coded 0, if no). Then, for each attitude object, we computed the percentage of respondents that generated a first response for each of the eight coded categories (within each object, the percentages across categories sum to 100%).

Data analytic strategy

Evaluation and Likert accessibility questions. We performed a preliminary analysis to assess differences in accessibility of love among the three attitude objects. Specifically, we performed a mixed ANOVA with attitude object (roses, chocolates, online dating product) as the within-subjects factor, and order of questionnaires (open-ended first or evaluations first) as the between-subjects factor. The dependent variable was the Likert accessibility measure. Sidak corrected pairwise comparisons were used to control for performing three tests (one for each pair of attitude objects). For the sake of comparison with the main study, we also examined evaluations of the attitude objects using the same analytic strategy.

We also examined whether respondents' first responses to the open-ended accessibility questions differed across the three attitude objects. Specifically, we used the non-parametric Cochran's *Q* test to determine whether the percentage of respondents' first responses, to the open-ended question, differed across roses, chocolates and the online dating product. Where Cochran's *Q* test was statistically significant, multiple comparisons were undertaken using the McNemar's test.

Assessing the relationship between accessibility of love and strength of positive evaluations. To test for the hypothesized relationship that accessibility of love is associated with stronger positive evaluation of attitude objects, we analyzed the data using mixed models. Mixed models account for interdependency among data points. It is beneficial because it allows us to model the relationship between accessibility of love and evaluation across the three attitude objects *within each person*, allowing each person to have a different slope. By assessing the relationship between accessibility of love and evaluation within each person, it effectively addresses third variable alternative hypotheses that affect between subjects regression and correlational analyses. We ran mixed models predicting attitude object evaluation from the Likert accessibility measure. Likert accessibility measure was treated as a random factor, and attitude object was included as a fixed factor. The intercept was also allowed to vary randomly as a function of respondent. Finally, our model included the

questionnaire order, as well as all two and three-way interactions between questionnaire order and Likert accessibility measure.

Mediation analysis. To examine the extent to which differences in the extent to which objects are associated with love mediates differences in evaluative judgments of objects, we conducted a within-subjects mediation analyses following procedures outlined by Judd, Kenny, and McClelland (2001). Specifically, we performed the mediation analysis twice: one, to examine the role of accessibility in explaining evaluative differences between roses and an online dating product, and a second time to examine the role of accessibility in explaining evaluative differences between a box of gift chocolates and an online dating product.

Within-subjects mediation analysis involves two steps. In step one, we regressed each evaluation measure on the accessibility measure from the same attitude object. In step 2, we regressed the evaluation difference between roses and online dating product on their accessibility sum and accessibility difference. Mediation is said to occur if 1) in step 1, accessibility is a significant predictor of evaluation for each attitude object, and if 2) in step 2, the difference in accessibility significantly predicts the difference in evaluation. Complete mediation is assumed to occur if in step 2, the intercept is no longer statistically significant. We centered the accessibility sum variable as recommended by Judd et al. 2001 so that the intercept represents the average accessibility effect.

Examining the role of demographic characteristics (age, gender, and relationship status). We examined the role of gender on evaluations and the Likert accessibility measure by repeating our previously stated analytic strategy. Specifically, we performed repeated measures ANOVA with attitude object as a within-subject factor (roses, chocolates, and online dating product), questionnaire order (evaluations first or Likert accessibility first), and gender as a categorical between-subject factors, along with all interactions. In case of significant interaction, we followed-up with independent t-tests and used Bonferroni corrected *p*-values for multiple tests. To test for the role of age, we used bivariate correlations to test the association of age and accessibility of the attitude objects.

To test for the role of relationship status, we followed exclusion criteria similar to the main study; we excluded respondents who selected the response “other” (1.5% of the sample; $n = 9$) for relationship status, as well as those who self-identified as “dating more than one person,” because this group had a considerably smaller sample size ($n = 12$) than the other four groups (*n*s > 75). This left a sample size of 591 for these analyses. We performed a repeated measures ANOVA with attitude object as a within-subject factor, questionnaire order (evaluations first or Likert accessibility first) and relationship status as a categorical between-subject variables along with all interactions. In the case of a statistically significant main effect of relationship status, we performed all pairwise comparisons using Tukey’s honestly significant difference (HSD). In case of significant interactions, we followed-up with pairwise comparisons and applied Bonferroni corrected *p*-values for multiple tests.

To examine whether gender, age and relationship status first response generated on the open-ended accessibility measure, we performed separate Chi square tests. Because we were interested only in the love/Valentine’s day category, these analyses focused only on this category of first response.

Results

Evaluations

The strength of positive evaluation significantly differed across the attitude objects (*F* (2, 610) = 1153. 41, $p < .001$, $\eta_p^2 = .66$). Chocolates (*M* = 4.17, *SD* = 1.01) were evaluated more positively than roses (*M* = 3.59, *SD* = 1.15; $p < .00001$), and roses were evaluated more positively than the online dating product (*M* = 1.70, *SD* = .98; $p < .00001$)[2].

Moreover, respondent characteristics (gender, age, relationship status) were related to evaluations of objects in a manner that was highly similar to the results observed in the main study. Compared to males, females evaluated roses ($t(610) = 7.684$, $p < .001$) and chocolates (t(610) = 4.935, $p < .001$) more positively, and, although not statistically significant, the online dating product more negatively ($t(610) = -1.224$, $p = .221$). Respondents' age was significantly correlated with the evaluation of roses ($r = .195$, $p < .001$), and the online dating site product ($r = .082$, $p = .047$), but not with chocolates ($r = -.058$, $p = .159$).

Finally, there was a significant interaction between relationship status and attitude object on evaluation of attitude objects ($F(6, 583)=7.605$, $p<.001$, $\eta_p^2=.038$). The mean evaluations as a function of object and relationship status, along with results of all pairwise comparisons, are shown in Figure S5. For evaluations of roses, the two groups of singles—i.e., interested in meeting someone and those not interested in dating—did not differ from each other, but reported less positive evaluations compared to respondents who identified as dating only one person and as partnership/married. For the online dating product, singles interested in meeting someone evaluated the online dating product more positively compared to each of the three other groups (all differences were significant at $p < .004$, except for dating only one person which was at $p < .052$).

Accessibility

Likert accessibility. The extent to which respondents reported that thoughts of love came to mind, as reflected by the Likert accessibility measure, significantly differed as a function of attitude object (*F* (2, 598) = 261.17, $p < .0001$, $\eta_p^2 = .30$; see panel A of Figure S6).[3] Roses more readily brought to mind thoughts of love compared to chocolates, $p < .0001$, and chocolates more readily brought to mind thoughts of love compared to the online dating product ($p < .002$).[4]

---

[2] The main effect of order of questionnaires was not significant (*F* (1, 610) = 2.763, $p = .09$, $\eta_p^2 = .005$). However, there was a statistically significant attitude object × order of questionnaire interaction (*F* (2, 610) = 4.75, $p = .009$, $\eta_p^2 = .008$). We ran follow-up tests to probe this two-way interaction. We found that the evaluation for roses was significantly (t (610) = 3.06, $p = .002$) more positive in the accessibility-first condition (M = 3.74, SD = 1.13) compared to the evaluation-first condition (M = 3.45, SD = 1.16). Mean evaluations between the two orders did not differ for chocolates (t (610) = 0.36, $p = .721$) or the online dating product (t (610) = 0.453, $p = .651$).

[3] The change in *df* of the analyses involving Likert accessibility is due to missing responses from 9 respondents for chocolates and 3 respondents for the online dating product.

[4] The main effect of order of questionnaires was also statistically significant (*F* (1, 598) = 4.97, $p = .02$, $\eta_p^2 = .008$). Specifically, for all three objects, accessibility of love was stronger in the accessibility-first condition compared to the

Accessibility of love did not significantly differ as a function of respondents' gender (all *p*s > .131). Likewise, respondents' age was not significantly correlated with the accessibility of roses (r = .000, p = .99), chocolates (r = -.044, p = .28), or the online dating site product (r = .077, p = .06). However, relationship status significantly interacted with attitude object (F(6, 572) = 3.914, p = .001, η_p^2 = .020). As shown in Figure S7, relationship status predicted the accessibility of love for roses (F(3, 587) = 11.084, p < .001, η_p^2 = .054), but it did not predict differences for chocolates (F(3, 580) = 1.829, p = .14, η_p^2=.009), or the online dating product (F(3, 579) = 2.234, p = .08, η_p^2 = .011). Singles not interested in dating reported that roses were more weakly associated with love, compared to each of the three other relationship status groups (*p*s < .020).

Open-ended accessibility. Table S8 shows the percentage of respondents who mentioned "love" or "Valentine's Day" or one of the other seven categories assessed using open-ended measure for the three attitude objects. Consistent with our assumptions, a higher percentage of respondents spontaneously mentioned "love" or "Valentine's Day" when viewing an image of roses (p < .001), compared to when viewing the image of the online dating product (see panel B of Figure S6). Also consistent with our assumptions, the percentage of respondents that spontaneously mentioned "relationships" in response to viewing the image of the online dating product was significantly higher compared to roses (p < .001), which was significantly higher compared to chocolates (p < .001). We did not find that viewing gift chocolates led respondents to spontaneously mention love or Valentine's Day more than when viewing the online dating product. This may be because positively valence objective features related to the appetitive nature of chocolates came to mind first (See Table S7).

Accessibility of love and Valentine's Day did not differ as a function of gender for roses (X^2 (1, N = 612) = 1.832, p = .176), chocolates (X^2 (1, N = 612) = 3.517, p =.061), or the online dating product (X^2 (1, N = 612) = 3.902, p =.048; higher than the Bonferroni adjusted p-value of .016). Accessibility of love also did not differ as a function of respondents' relationship status (*p*s > .214) or age (*p*s >.395).

Does greater accessibility of love predict stronger positive evaluations of attitude objects?

Between-person analyses. For each of the three attitude objects, the more strongly the object was associated with the concept love, the more positive were respondents' evaluative judgments (see Table S9). The correlation between accessibility of love and positivity of evaluation occurred for roses (r = .40, p < .01) and chocolates (r = .30, p = .01). It is worth noting that stronger associations between the online dating product and love also predicted more positivity towards the online dating product (r = .49, p < .01). This finding is consistent with the idea that the concept of love is positive and that to the extent that any object is associated with love, it will be evaluated more positively.[5]

Within-person analyses. Could the observed relationship between accessibility of love and strength of positive evaluations simply reflect a general disposition to perceive and evaluate items positively? For example, someone with a proclivity to view the world favorably might report both heightened accessibility of positive concepts, such as love, and evaluate objects more positively. To

evaluation-first condition. There was no statistically significant interaction between attitude objects and order of questionnaires (F (2, 598) = .94, p = .38, η_p^2 = .002).

[5] The results of three linear regressions, which included the effect of order of questionnaires, led to the similar conclusions. Accessibility significantly predicted evaluation for roses (b = .27, p < .01), chocolates (b = .22, p < .01), and the online dating product (b = .27, p < .01). There were no main effects of, or interactions with, order of questionnaire in any of the models (all *p*s >.06).

address this issue with our current data, we used mixed models. MLM is able to assess the association between accessibility of love and evaluation of attitude objects *within each person*. By doing so, the effect of person is held constant, effectively addressing third variable alternative hypotheses.

We found a robust within-person effect such that greater accessibility of the concept love was associated with stronger positive evaluations (b = .21, SE = .02, p < .0001). In other words, within a *given* respondent, the more that the attitude object was associated with the concept love, the more positively the object was evaluated.[6][7] These findings provide stronger evidence that objects that are associated with love are judged more positively.

Mediation analyses. Finally, we examined whether differences in the accessibility of love between attitude objects (roses being judged more favorably than the online dating product) mediated differences in evaluations. We followed procedures outlined by Judd et al. (2001; see Data analytic section for details) and report the results from both, step 1 and step 2, of the mediation analyses in Table S10. In short, we found evidence that differences in the extent to which objects were associated with the concept love (as indicated by the Likert accessibility measure) partially mediated differences in the evaluative judgments of the objects. Specifically, the difference in accessibility between roses and the online dating product was a significant predictor of the difference in evaluation, and similarly the difference in accessibility of love between chocolates and the online dating product was a significant predictor of the difference in their evaluation. In both cases, the intercept remained statistically significant, indicating partial mediation (see Table S10). Lastly, the difference in accessibility of love between roses and chocolates was also a significant predictor of the difference in their evaluation. The intercept was again significant, suggesting partial mediation.

Conclusion

The first aim of the supplemental study was to examine whether an image of red roses, and of a box of gift chocolates, are more strongly associated with love and Valentine's Day, compared to an image of an online dating product. Consistent with intuitions, red roses and gift chocolates were more strongly associated with love, as assessed with the quantitative Likert measure of accessibility, than the online dating product, and that people were more likely to spontaneously mention love or Valentine's Day upon viewing an image of red roses, compared to the online dating product and chocolates. Respondents were not more likely to spontaneously mention love or Valentine's Day after viewing an image of gift chocolates, but this may reflect that other aspects of the object, in particular its appetitive nature, were more salient.

---

[6] There was also a statistically significant two-way interaction between accessibility of love and attitude object (F (2, 1523) = 11.37, p < .001). Follow-up tests showed that the relationship between accessibility and evaluation was stronger for the online dating product, compared to roses (accessibility × object: b = .08, SE = .03, p = .016), and stronger for roses compared to chocolates (accessibility × object: b = -1.83, SE = .03, p < .0001). These results converge with the results from the zero-order correlations between accessibility and evaluation for each attitude object reported in Table S8.

[7] Similar to the results of the mixed ANOVA reported in Footnote 2, the two-way interaction between attitude object and order of questionnaire was statistically significant (F (2, 1444) = 3.77, p = .02). Follow-up tests showed that the positive evaluation of roses was greater in the accessibility-first condition compared to the evaluation-first condition. The two-way interaction between Likert accessibility and questionnaire order (F (1, 1661) = .94, p = .33) and the three-way interaction between accessibility, attitude object, and questionnaire order were not statistically significant (F (2, 1523) = 2.24, p = .10).

More importantly, the supplemental study aimed to provide support for a key assumption in our reasoning, namely that the stronger an object's associations with love, the more positively the object was evaluated. Using between-subjects analyses, within-subjects analyses that completely control for person characteristics, and mediation analysis, we show that objects that are more strongly associated with love are judged more favorably. Past work has shown that love is represented as a network with positive evaluative links (Kent, 2010), and that priming love activates abstract and specific memories (Wlododarski & Dunbar, 2014). Here we show that the accessibility of the concept love is a predictor of the positivity of evaluative judgments of objects.

Collectively, these findings provide a plausible mechanism for why roses and chocolates, more so that the online dating product, increase in positivity as Valentine's Day approaches. Red roses and gift chocolates are more likely to be associated with love. Thus, as Valentine's Day draws near and the cultural relevance of love symbols increase, the *roses—love* and *chocolates—love* associations become more salient. Because the concept love itself is hugely positive, this in turn results in heightened positive evaluations for objects associated with love. Online dating is not viewed as a symbol of love. Thus, it does not show the same increase in positivity as Valentine's Day nears.

2 References

Ayers, J. W., Althouse, B. M., Allem, J., Rosenquist, J. N., & Ford, D. E. (2013). Seasonality in seeking mental health information on Google. *American Journal of Preventive Medicine, 44(5)*, 520-525.

Bargh, J. A., Lombardi, W. J., & Higgins, E. T. (1988). Automaticity of chronically accessible constructs in person X situation effects on person perception: It's just a matter of time. *Journal of Personality and Social Psychology, 55,* 599-605.

Choi, H., & Varian, H. (2012). Predicting the present with Google Trends. *The Economic Record, 88*, 2-9.

Cooper, C. P., Mallon, K. P., Leadbetter, S., Pollack, L. A., & Peipins, L. A. (2005). Cancer Internet search activity on a major search engine, United States 2001 – 2003. *Journal of Medical Internet Research, 7,* e36.

Ettredge, M., Gerdes, J., Karuga, G. (2005). Using web-based search data to predict macroeconomics statistics. *Communications of the ACM, 48(11),* 87-92.

Fazio, R. H., Williams, C. J., & Powell, M. C. (2000). Measuring associative strength: Category-item associations and their activation from memory. *Political Psychology, 21*, 7-25.

Ginsberg, J., Mohebbi, M. H., Patel, R. S., Brammer, L., Smolinski, M. S., Brilliant, L. (2009). Detecting influenza epidemics using search engine query data. *Nature, 457,* 1012-1014.

Hazan, C., & Shaver, P. (1987). Romantic love conceptualization as an attachment process. *Journal of Personality and Social Psychology, 52,* 511-524.

Higgins, E. T., King, G. A., & Mavin, G. H. (1982). Individual construct accessibility and subjective impressions and recall. *Journal of Personality and Social Psychology, 43,* 35-47.

Judd, C. M., Kenny, D. A., & McClelland, G. H. (2001). Estimating and testing mediation and moderation in within-subject designs. *Psychological Methods, 6,* 115-134.

Kent, S. (2010). Symbols of Love: Consumption, Transnational Migration, And The Family In San Salvador, El Salvador. *Urban Anthropology and Studies of Cultural Systems and World Economic Development, 39,* 73-108.

MacInnis, C. C., & Hodson, G. (2015). Do American states with more religious or conservative populations search more for sexual content on Google? *Archives of Sexual Behavior, 44,* 137-147.

Polgreen, P. M., Chen, Y., Pennock, D. M., Nelson, F. D. (2008). Using internet searches for influenza surveillance. *Clinical Infectious Diseases, 47,* 1441-1448.

Wanous, J. P., Reichers, A. E., & Hudy, M. J. (1997). Overall job satisfaction: How good are single-item measures? *Journal of Applied Psychology, 82,* 247-252.

Wlododarski, R., & Dunbar, R. I. M. (2014). The effects of romantic love on mentalizing abilities. *Review of General Psychology, 18,* 313-321.

3 Supplementary Figures and Tables

3.1 Supplementary Figures

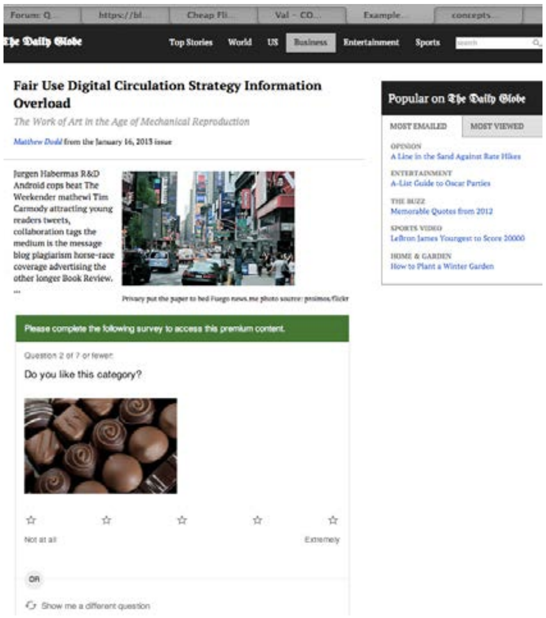

Figure S1. Screenshot of survey as it appears to respondents completing the survey on a computer.

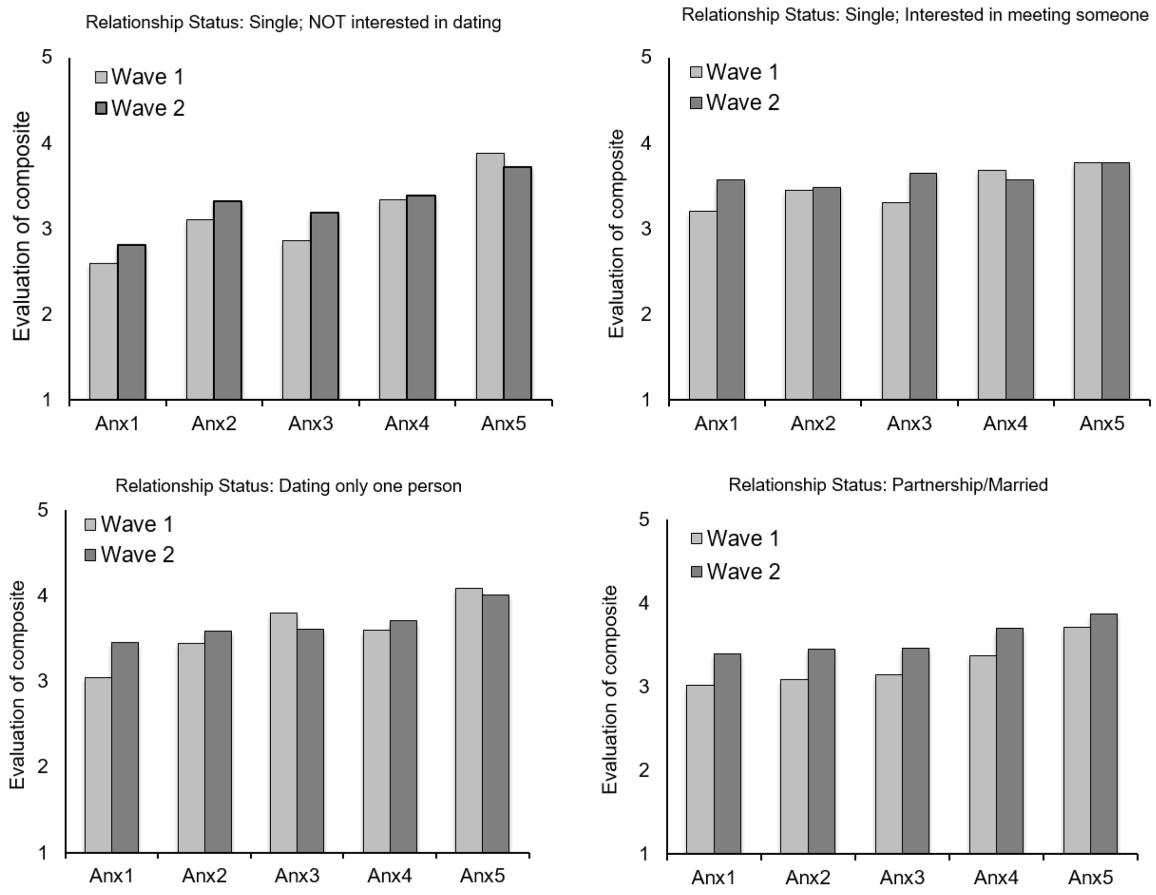

Figure S2. Mean evaluations (higher scores reflect greater positivity) of love symbols (composite score of roses and chocolates) as a function of relationship status and adult attachment anxiety. Each panel represents the data for one of the relationship status groups: (a) single and not interested in dating; (b) single and interested in dating; (c) dating one person; and (d) married/partnership. The y-axis represents the mean evaluation of love symbols (composite score representing the average of evaluation of roses and evaluations of chocolates) and the x-axis represents responses on the particular attachment question. For the sake of presenting all the data, we graphed responses to each question as nominal levels, ranging from 1 (*not at all*) to 5 (*extremely*).

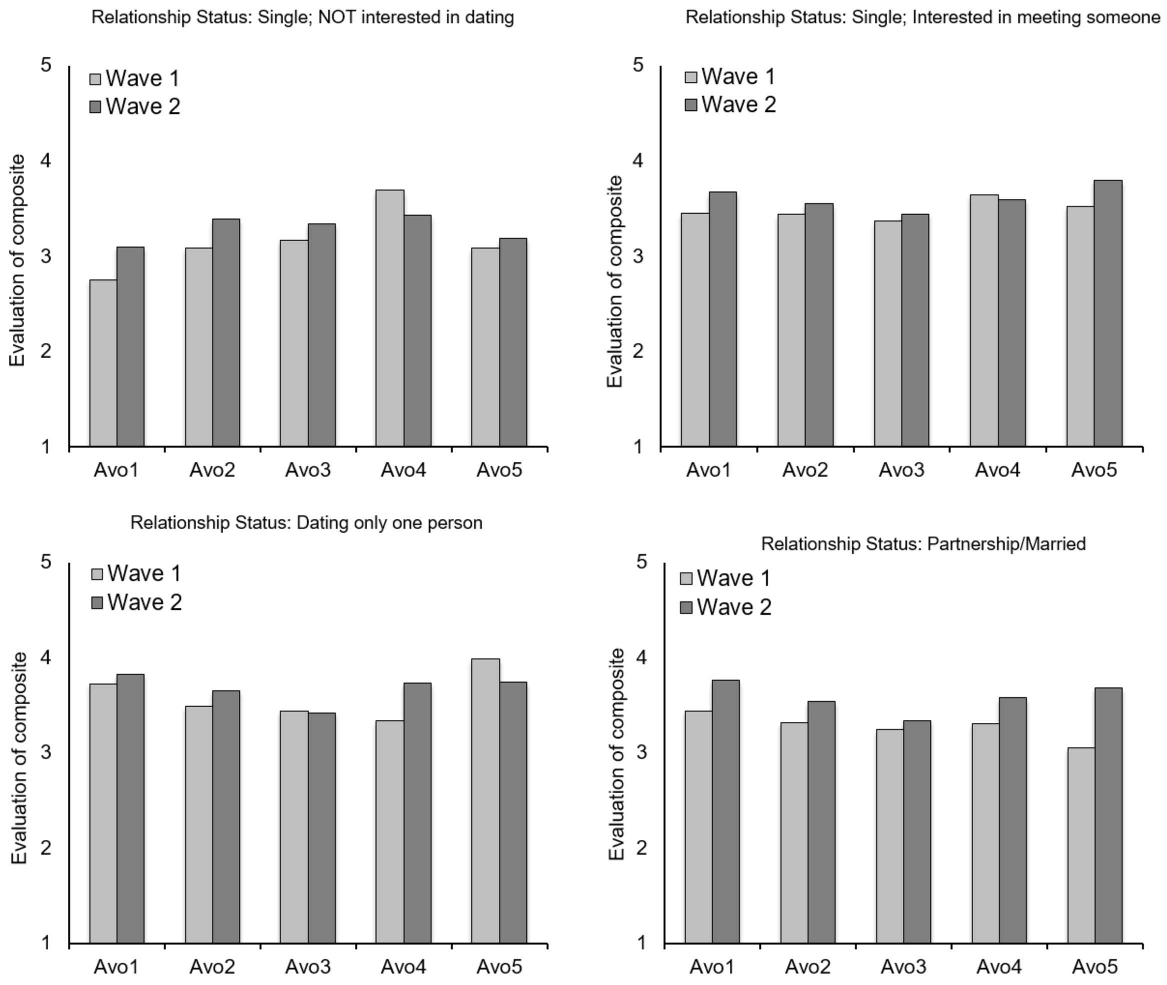

Figure S3. Mean positive evaluations of love symbols (composite score of roses and chocolates) as a function of relationship status and adult attachment avoidance. Each panel represents the data for one of the relationship status groups: (a) single and not interested in dating; (b) single and interested in dating; (c) dating one person; and (d) married/partnership. The y-axis represents the mean evaluation of love symbols (composite score representing the average of evaluation of roses and evaluations of chocolates) and the x-axis represents responses on the particular attachment question. For the sake of presenting all the data, we graphed responses to each question as nominal levels, ranging from 1 (*not at all*) to 5 (*extremely*).

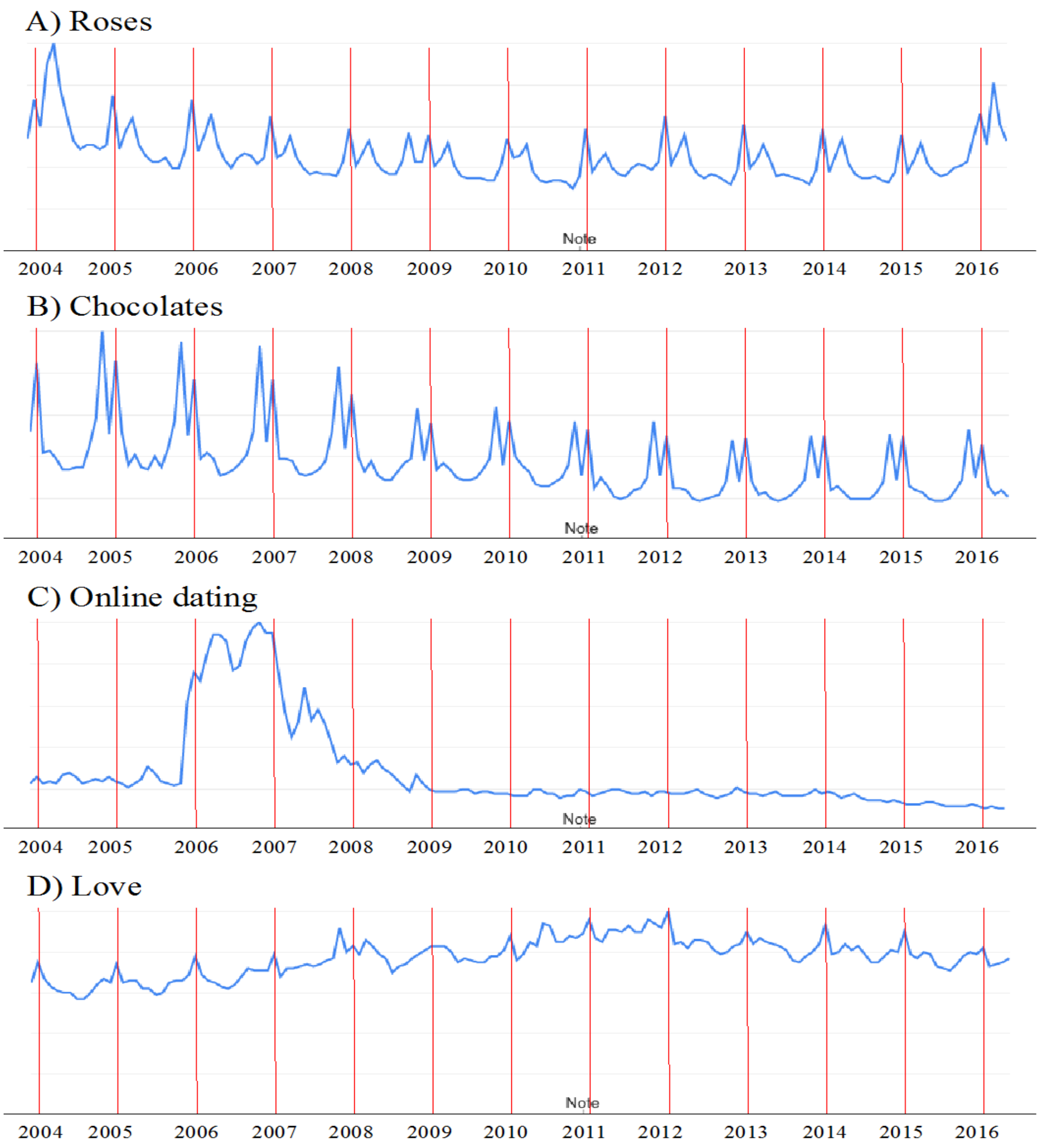

Figure S4. Population-level Google search term frequency for “roses” (panel a), “chocolates” (panel b), “online dating” (panel c), and “love” (panel d) as a function of day. In each panel, the red vertical lines on the x-axis indicate Valentine’s Day (February 14th) for the given year. Google search term frequency is a relative measure based on the total number of Google searches for the particular term specified in the U.S. during the time period being examined. The maximum query frequency in the time period specified is standardized to 100, and the query frequency at the initial date being examined is standardized to be zero by Google Trends. Thus, the absolute values of the search frequency are not interpretable; only the relative changes across time within a Google Trends query are interpretable.

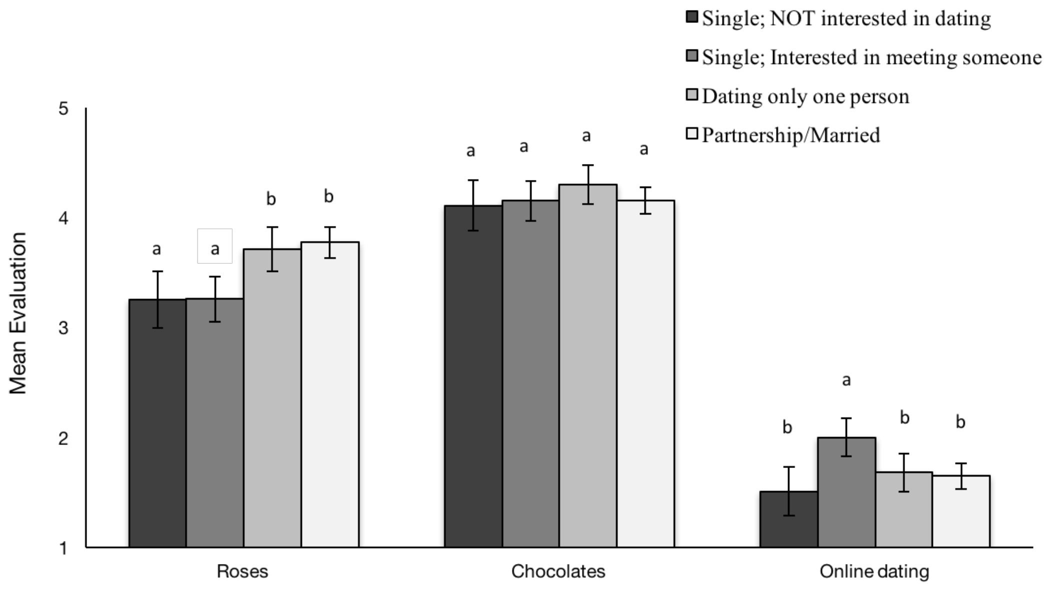

Fig. S5. Mean evaluation of attitude objects (roses, chocolates, and an online dating product) as a function of relationship status (single and not interested in dating; single and interested in dating; dating one person; married/partnership). Evaluation was measured on a Likert scale from 1 (*not at all*) to 5 (*extremely*). Higher numbers reflect greater positivity. Error bars represent 95% CI. For comparisons between relationship status groups within each attitude object, means sharing the same letter are not significantly different according to Tukey's HSD test.

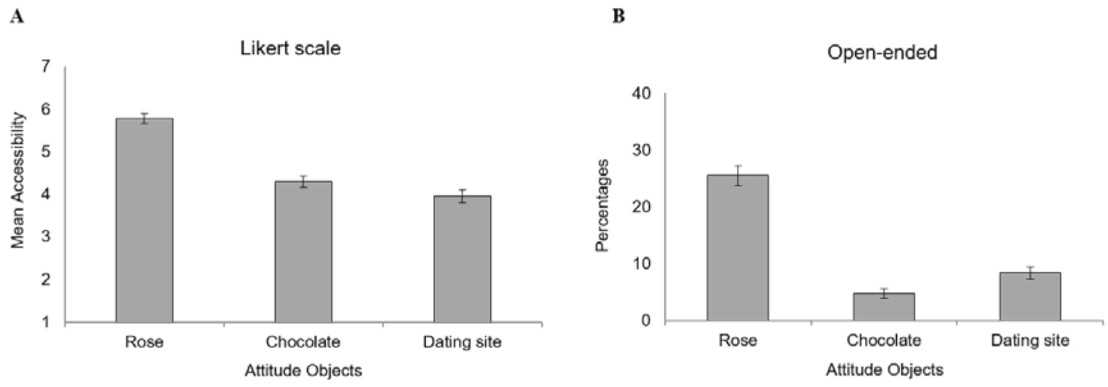

Figure S6. Mean accessibility of the concept love assessed on a *Likert scale* from 1 (*not at all*) to 7 (*very much*) (panel A) and percentage of respondents who spontaneously mentioned "love" or "Valentine's Day" as their first response (panel B), assessed using *open-ended measure*, for the three attitude objects. Error bars represent 95% CI.

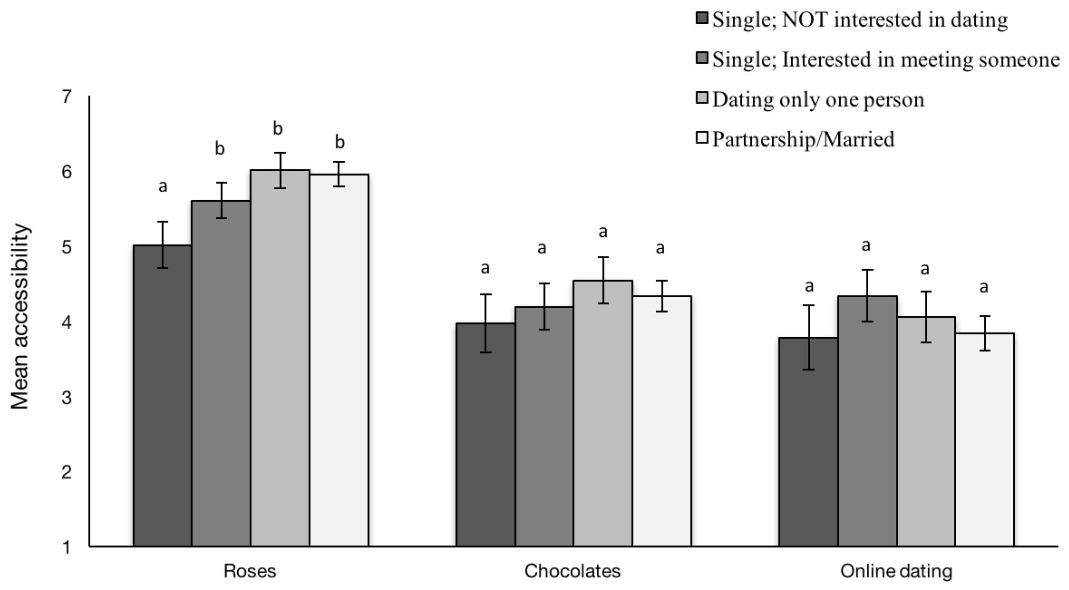

Fig. S7. Mean accessibility of the concept love for the three attitude objects (roses, chocolates, and online dating product) as a function of relationship status (single and not interested in dating; single and interested in dating; dating one person; married/partnership). Accessibility was measured on a Likert scale from 1 (*not at all*) to 7 (*very much*). Higher numbers reflect greater accessibility. Error bars represent 95% CI. Bars sharing the same letter are not significantly different according to Tukey's HSD test.

3.2 Supplementary Tables

Table S1

Results of ANOVAs examining the effect of Valentine's Day on evaluations of attitude objects (roses, chocolates, and online dating product), statistically controlling for all procedural variables.

Procedural variable	*Item*	*df*	*F*	*p*	η_p^2
Wave	Roses	1	67.85	.001	.005
	Chocolate	1	70.85	.001	.005
	Dating Site	1	5.48	.019	.000
Question wording	Roses	1	4.82	.028	.000
	Chocolate	1	.00	.960	.000
	Dating site	1	.01	.923	.000
Question order	Roses	1	8.92	.003	.001
	Chocolate	1	32.61	.000	.002
	Dating site	1	.28	.597	.000
Platform	Roses	1	274.80	<.001	.018
	Chocolate	1	355.38	<.001	.023
	Dating site	1	155.65	<.001	.010
Day of the week	Roses	4	5.77	<.001	.002
	Chocolate	4	9.99	<.001	.003
	Dating site	4	4.48	.001	.001
Hour of the day	Roses	1	5.70	.017	.000
	Chocolate	1	12.61	<.001	.001
	Dating site	1	11.93	.001	.001

df error = 14783. *Note.* Wave = Temporal proximity to Valentine's Day (Wave 1 (Feb. 3-7) vs. Wave 2 (Feb. 12-14)). Question wording = Wording of evaluation question ("*Do you like this category?*" vs. "*Do you like this category of product*"?). Order of survey questions = order in which the evaluation questions were completed (evaluation questions first vs. after attachment questions). *Platform* = technology used to complete the survey (Android vs. desktop). All variables were entered as categorical covariates except for hour of the day, which was entered as a continuous variable.

Table S2

Mean evaluations of attitude objects (roses, chocolates, and online dating product) as a function of temporal proximity to Valentine's Day (wave 1 vs. wave 2) for each gender separately.

		Wave 1		Wave 2		Test of gender × wave		
						df	*F*	*p*
Roses	*M*	3.30	2.75	3.66	3.07	1	.90	.344
	SD	1.33	1.36	1.26	1.29			
	n	1319	1793	4334	5603			
Chocolates	*M*	3.60	3.16	3.86	3.52	1	3.05	.081
	SD	1.36	1.42	1.28	1.33			
	n	1319	1793	4334	5603			
Online dating	*M*	1.58	1.65	1.68	1.72	1	.23	.630
	SD	.99	1.02	1.05	1.06			
	n	1319	1793	4334	5603			

df error = 13045. *Note.* Not all respondents had gender information (see Method section). Evaluations were made on a scale from 1 (*not at all*) to 5 (*extremely*). Higher numbers reflect greater positivity.

Table S3

Results of ANOVAs examining effects of temporal proximity to Valentine's Day, relationship status, adult attachment anxiety, and adult attachment avoidance on evaluation of love symbols (top half of table) and online dating product (bottom half of table) controlling for procedural variables.

Evaluation of love symbols (composite of roses and chocolates)				
Source	*df*	*F*	*p*	η_p^2
Status	3	87.68	.001	.018
Wave	1	52.01	.001	.004
Anxious attachment	1	218.39	.001	.015
Avoidant attachment	1	4.25	.039	.000
RS × Wave	3	.91	.435	.000
Anxious attachment × Wave	1	14.56	.001	.001
Avoidant attachment × Wave	1	2.13	.144	.000
Status × Anxious attachment × Wave	6	7.67	.001	.003
Status × Avoidance attachment × Wave	6	7.92	.001	.003
Evaluation of online dating site product				
Source	*df*	*F*	*p*	η_p^2
Status	3	28.54	.001	.006
Wave	1	6.60	.01	.000
Anxious attachment	1	200.75	.001	.014
Avoidant attachment	1	78.08	.001	.005
Status × Wave	3	1.11	.344	.000
Anxious attachment × Wave	1	.26	.608	.000
Avoidant attachment × Wave	1	2.14	.143	.000
Status × Anxious attachment × Wave	6	9.79	.001	.004
Status × Avoidance attachment × Wave	6	1.68	.12	.001

df error =14266. *Note.* Evaluation of love symbols was the mean of evaluations of roses and chocolates. Evaluations were made on a scale from 1 (*not at all*) to 5 (*extremely*). Higher numbers reflect greater positivity. RS = Relationship status (single and not interested in dating; single and interested in dating; dating one person; married/partnership); Wave = Temporal proximity to Valentine's Day (Wave 1 (Feb. 3-7) vs. Wave 2 (Feb. 12-14)). The ANOVAs included all procedural covariates. The results were highly similar when analyses were performed not statistically controlling for procedural variables.

Table S4

Tests of interaction between respondent age and temporal proximity to Valentine's Day (wave 1 vs. wave 2) on evaluations of attitude objects (roses, chocolates, and online dating product).

	df	F	p	η_p^2
Roses	1	8.184	0.004	0.001
Chocolates	1	0.088	0.767	0.000
Dating Site	1	1.328	0.249	0.000

df error = 12295. *Note.* Not all respondents had age information (see Method section).

Table S5

Mean evaluations of attitude objects (roses, chocolates, and online dating product) as a function of temporal proximity to Valentine's Day (wave 1 vs. wave 2) for each age group separately.

		Wave 1			Wave 2			Effect of wave	
Age range		*M*	*n*	*SD*	*M*	*n*	*SD*	*F*	*p*
18-24	Roses	3.39	439	1.29	3.42	1692	1.26	0.16	0.68
	Chocolates	3.83	439	1.27	3.92	1692	1.23	1.54	0.21
	Online dating	1.71	439	1.06	1.73	1692	1.04	0.21	0.65
25-34	Roses	2.95	542	1.36	3.35	2163	1.26	41.62	<.001
	Chocolates	3.38	542	1.35	3.81	2163	1.22	50.89	<.001
	Online dating	1.65	542	1.01	1.81	2163	1.1	9.44	.002
35-44	Roses	2.97	432	1.42	3.31	1760	1.31	22.82	<.001
	Chocolates	3.22	432	1.41	3.63	1760	1.34	31.27	<.001
	Online dating	1.53	432	0.97	1.7	1760	1.02	9.52	.002
45-54	Roses	2.70	511	1.36	3.32	1490	1.35	79.56	<.001
	Chocolates	3.05	511	1.49	3.55	1490	1.37	47.54	<.001
	Online dating	1.58	511	0.95	1.67	1490	1.04	2.52	0.11
55-64	Roses	2.94	592	1.32	3.26	1447	1.35	23.64	<.001
	Chocolates	3.21	592	1.36	3.47	1447	1.38	14.61	<.001
	Online dating	1.59	592	0.97	1.62	1447	1.04	0.49	0.48
65+	Roses	3.00	357	1.41	3.4	874	1.34	21.32	<.001
	Chocolates	3.3	357	1.45	3.53	874	1.35	7.45	.006
	Online dating	1.62	357	1.02	1.63	874	1.02	0.07	0.80

Note. Not all respondents had age information (see Method section). Evaluations were made on a scale from 1 (*not at all*) to 5 (*extremely*). Higher numbers reflect greater positivity.

Table S6

Demographic characteristics of respondents (gender, age, relationship status, divorce status, income, urban density, and geographic region).

Demographic characteristic	*n*	%
Gender		
Females	315	51.5
Males	297	48.5
Relationship Status		
Single; Not interested in dating	75	12.3
Single; Interested in meeting someone	120	19.6
Dating only one person	125	20.4
Dating more than one person	12	2
Partnership/Married	271	44.3
Other	9	1.5
Divorce Status		
No	534	87.3
Yes	78	12.7
Urban Density		
Urban	209	34.2
Suburban	297	48.5
Rural	106	17.3
Geographic Region		
Midwest	140	22.9
Northeast	168	27.5
South	179	29.2
West	125	20.4

N = 612

Table S7

Examples of respondents' actual first responses coded into each of the nine categories for the three attitude objects.

Categories	Roses	Chocolates	Online
Love/ Valentine's Day	Love/	Love/ Valentine's	Love
Relationships	Wife	-	Couple
Objective features	Red	Dark	Online
Gifts	Bouquet	Gift	-
Commercialism	Expensive	-	Commercials
Positive evaluation	I love roses	I like chocolate	Cute
Negative evaluation	Hate roses	I don't like	Worst
Other	Florist	Have so much	Too single

Note. For chocolates, there were no responses coded into the Relationships or Commercialism categories. For dating product, no responses were coded into the Valentine's Day or Gifts categories.

Table S8

Percentage of respondents who mentioned "love" or "Valentine's Day" (or one of seven other categories) assessed using open-ended measure for the three attitude objects (roses, chocolates, online dating product).

Categories	Roses		Chocolates		Dating product	
	M	*SD*	*M*	*SD*	*M*	*SD*
Love/Valentine's Day	25.49_a	43.61	4.74_b	21.26	8.33_c	27.66
Relationship	0.98_a	9.86	0.00_b	0.00	36.27_c	48.11
Objective features	47.71_a	49.98	54.90_b	49.80	22.88_c	42.03
Gifts	0.65_a	8.06	0.49_a	6.99	0.16_a	4.04
Commercialism	0.65_a	8.06	0.16_a	4.04	5.23_b	22.27
Positive evaluation	21.57_a	41.16	33.82_b	47.35	3.10_c	17.35
Negative evaluation	2.12_a	14.43	3.76_a	19.03	21.41_b	41.05
Other	0.82_a	9.00	$2.12_{a,b}$	14.43	2.61_b	15.96

Note. For each first descriptor category (i.e., within each row), percentages with different subscripts differ significantly at $p < .016$ (Bonferroni corrected p value).

Table S9

Zero-order correlations between evaluations and accessibility of love for attitude objects (roses, chocolates, and an online dating product).

	Evaluation			Accessibility		
	Roses	Chocolates	Online Dating	Roses	Chocolates	Online Dating
Evaluation						
Roses		0.34**	0.14*	0.40**	0.30**	0.04
Chocolates			0.10*	0.12**	0.30**	0.06
Online dating				0.15**	0.20**	0.49**
Accessibility						
Roses					0.41**	0.22**
Chocolates						0.15**
Online dating						

Note. * $p < .05$. ** $p < .01$.

Table S10

Results of mediation analyses examining the role of accessibility on evaluations.

	Step 1					*Step 2*			
	b	SE	*t*	*p*		*b*	SE	*t*	*p*
Predictor					*Predictor*				
Intercept	1.65	.18	8.76	< .0001	Intercept	1.37	.07	19.27	< .0001
Accessibility	.34	.03	10.63	< .0001	Diff (roses – online dating)	.28	.03	10.57	< .0001
Predictor					Sum (roses + online dating)	.02	.02	1.16	= .247
Intercept	3.39	.11	31.76	< .0001		*b*	SE	*t*	*p*
Accessibility	.18	.02	7.74	< .0001	*Predictor*				
Predictor					Intercept	2.40	.05	46.21	< .0001
Intercept	.77	.08	9.41	< .0001	Diff (choco – online dating)	.16	.02	7.30	< .0001
Accessibility	.23	.02	12.61	< .0001	Sum (choco + online dating)	-.06	.02	-3.00	= .003
					Predictor				
					Intercept	-.84	.07	-12.74	< .0001
					Diff (roses – choco)	.18	.03	6.02	< .0001
					Sum (roses + choco)	.11	.02	5.62	< .0001

Note. Within subjects mediation analysis (Judd et al. 2001) involves two steps. In step one, we regressed each evaluation measure on the accessibility measure from the same attitude object. In step 2, we regressed the evaluation difference between roses and the online dating (and in a separate analysis between chocolates and the online dating product) product on their accessibility sum and accessibility difference.

Mediation is said to occur if 1) in step 1, accessibility is a significant predictor of evaluation for each attitude object, and if 2) in step 2, the difference in accessibility significantly predicts the difference in evaluation. Complete mediation is assumed to occur if in step 2, the intercept is no longer statistically significant. We centered the accessibility sum variable as recommended by Judd et al. 2001 so that the intercept represents the average accessibility effect. In the present analyses, differences in the evaluation of roses and the online dating product, and in the evaluation of chocolates and the online dating product, are explained in part by differences (respectively, roses vs. online dating product, and chocolates vs. online dating product) in the accessibility of love.

Appendix

- Figures S8 to S20 showing Google web search frequencies plotted for roses (top panel), chocolates (second panel), online dating (third panel), and love (fourth panel) for the months of January to February for each year, 2004-2016.
- Figures S21 to S24 to show the Google web search frequencies plotted for multiple terms entered simultaneously in the Google Trends query for all available years (2004-2016).

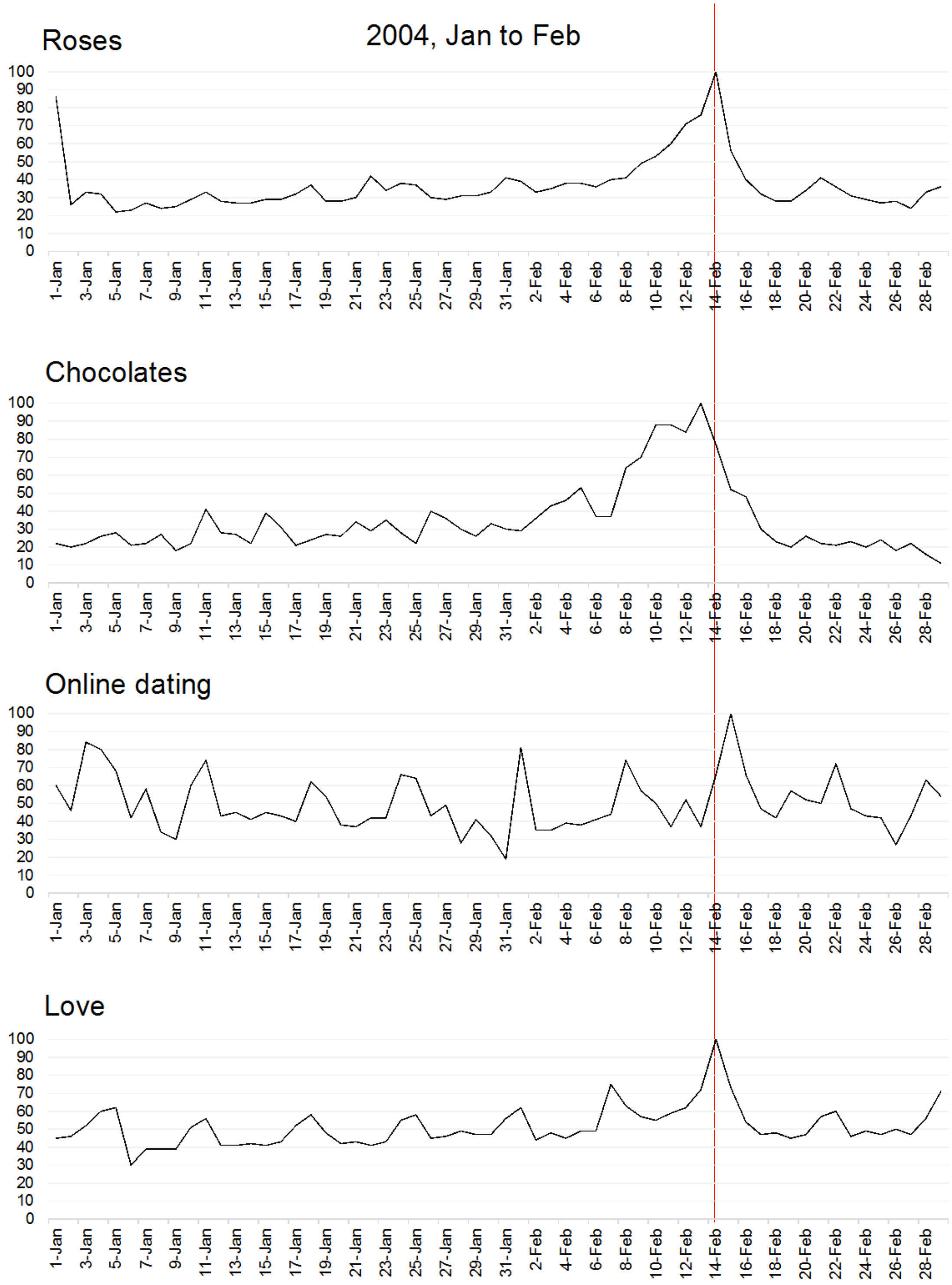

Figure S8. Population-level Google search term frequency for “roses” (panel a), “chocolates” (panel b), “online dating” (panel c), and “love” (panel d) between January and February, 2004. In each panel, the red vertical lines on the x-axis indicate Valentine’s Day (February 14th) for the given year.

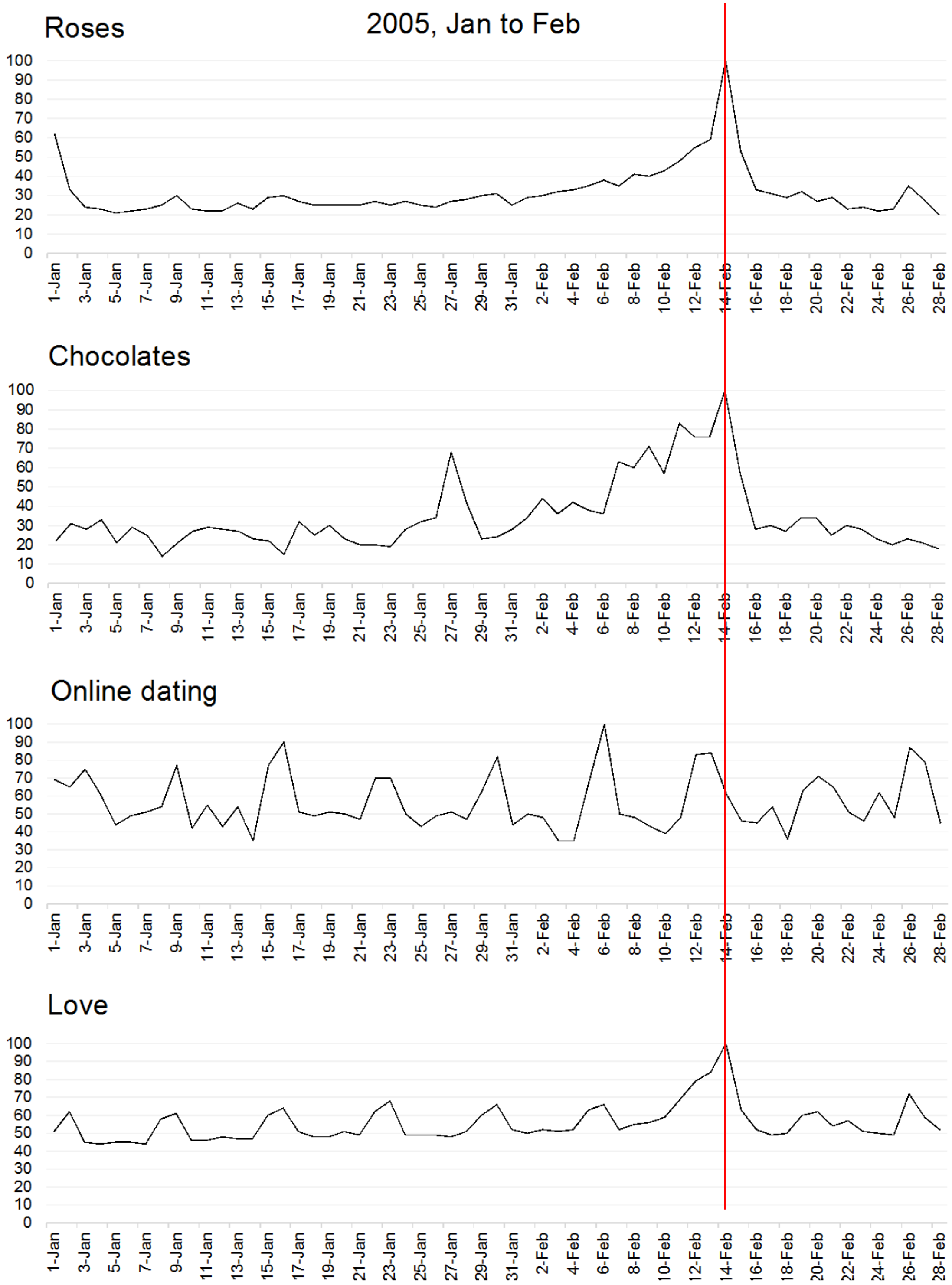

Figure S9. Population-level Google search term frequency for “roses” (panel a), “chocolates” (panel b), “online dating” (panel c), and “love” (panel d) between January and February, 2005. In each panel, the red vertical lines on the x-axis indicate Valentine’s Day (February 14th) for the given year.

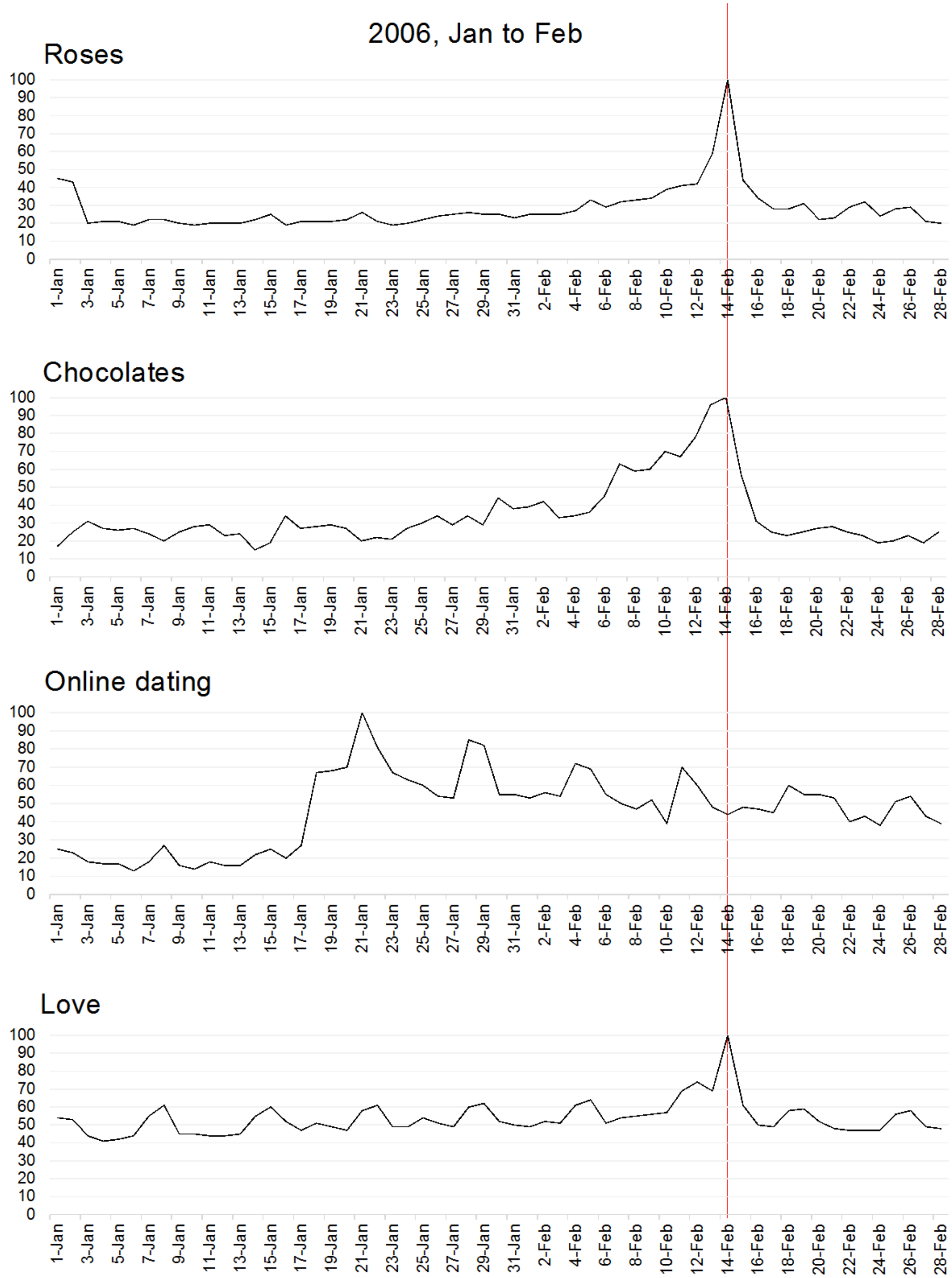

Figure S10. Population-level Google search term frequency for "roses" (panel a), "chocolates" (panel b), "online dating" (panel c), and "love" (panel d) between January and February, 2006. In each panel, the red vertical lines on the x-axis indicate Valentine's Day (February 14th) for the given year.

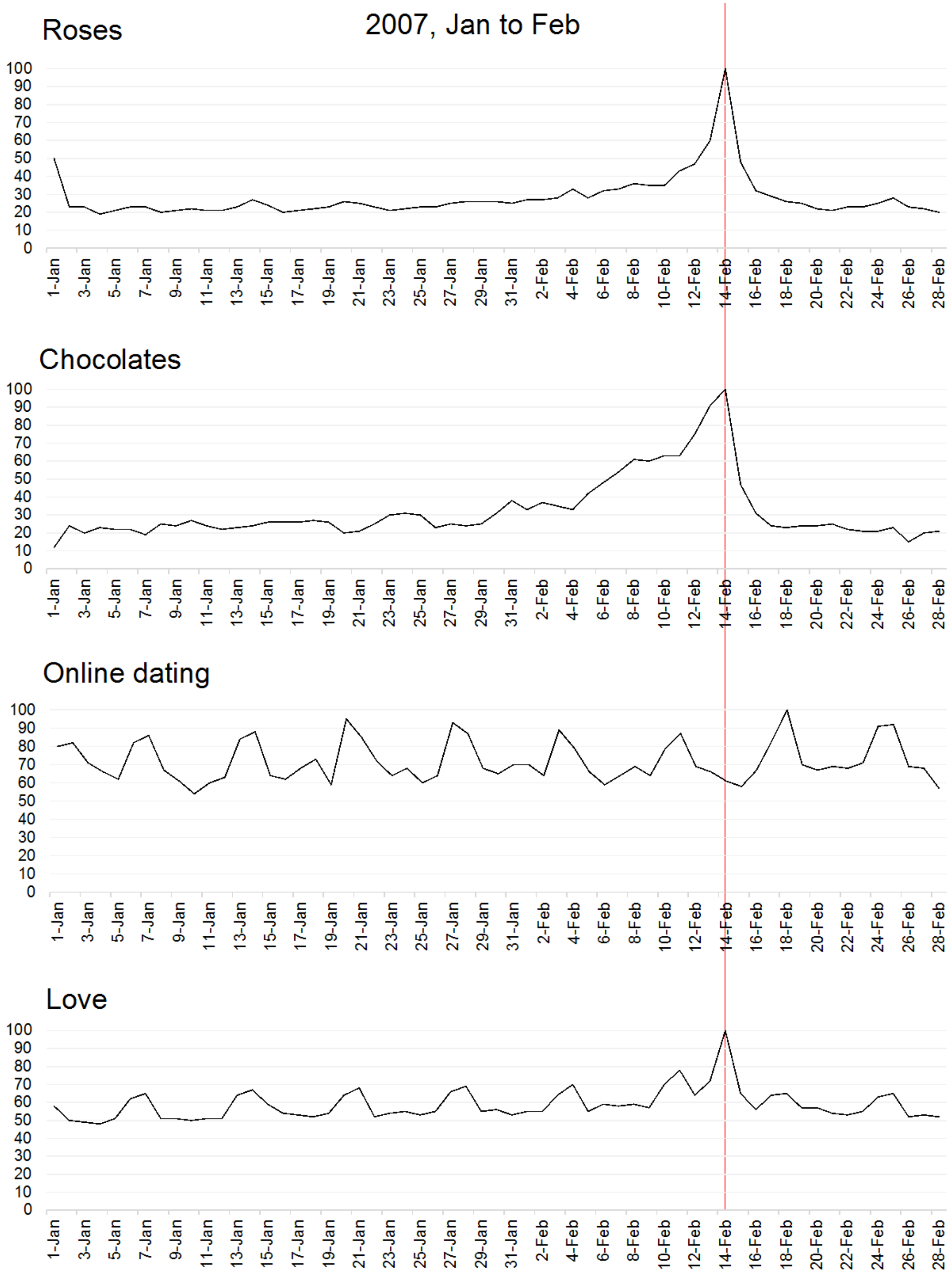
2007, Jan to Feb
Roses
Chocolates
Online dating
Love
100
90
80
70
60
50
40
30
20
10
0
1-Jan
3-Jan
5-Jan
7-Jan
9-Jan
11-Jan
13-Jan
15-Jan
17-Jan
19-Jan
21-Jan
23-Jan
25-Jan
27-Jan
29-Jan
31-Jan
2-Feb
4-Feb
6-Feb
8-Feb
10-Feb
12-Feb
14-Feb
16-Feb
18-Feb
20-Feb
22-Feb
24-Feb
26-Feb
28-Feb

Figure S11. Population-level Google search term frequency for "roses" (panel a), "chocolates" (panel b), "online dating" (panel c), and "love" (panel d) between January and February, 2007. In each panel, the red vertical lines on the x-axis indicate Valentine's Day (February 14th) for the given year.

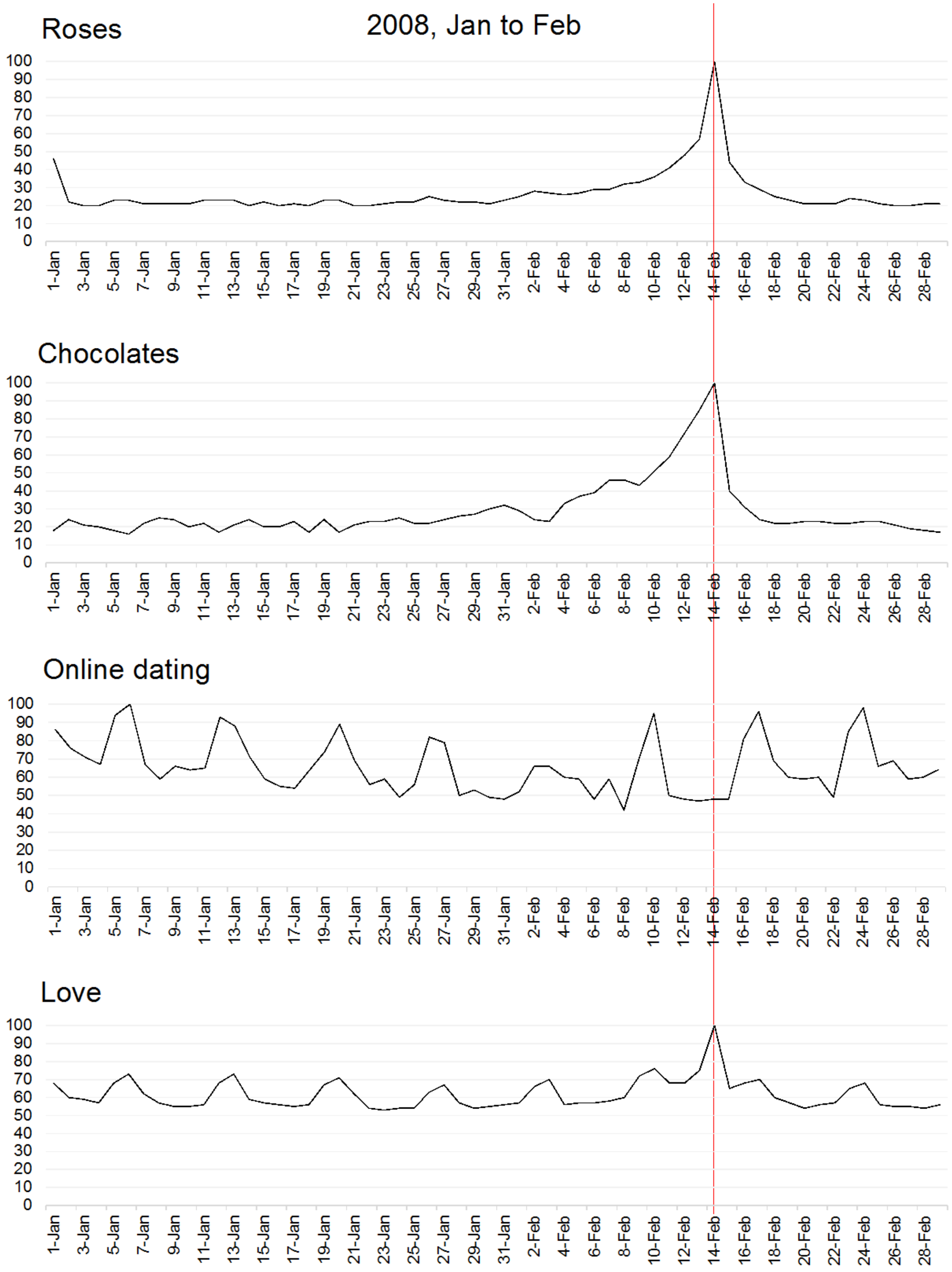

Figure S12. Population-level Google search term frequency for "roses" (panel a), "chocolates" (panel b), "online dating" (panel c), and "love" (panel d) between January and February, 2008. In each panel, the red vertical lines on the x-axis indicate Valentine's Day (February 14th) for the given year.

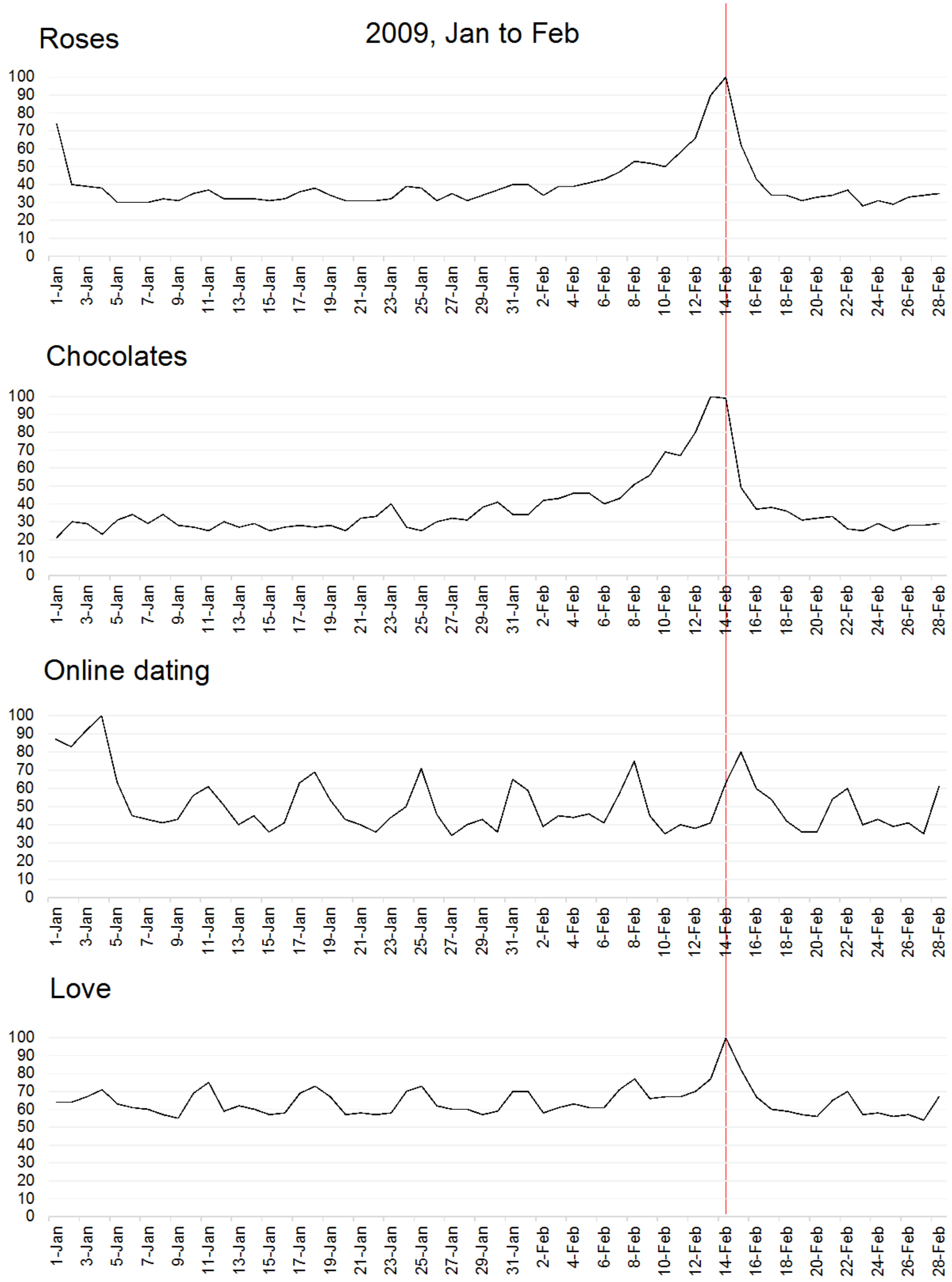

Figure S13. Population-level Google search term frequency for "roses" (panel a), "chocolates" (panel b), "online dating" (panel c), and "love" (panel d) between January and February, 2009. In each panel, the red vertical lines on the x-axis indicate Valentine's Day (February 14th) for the given year.

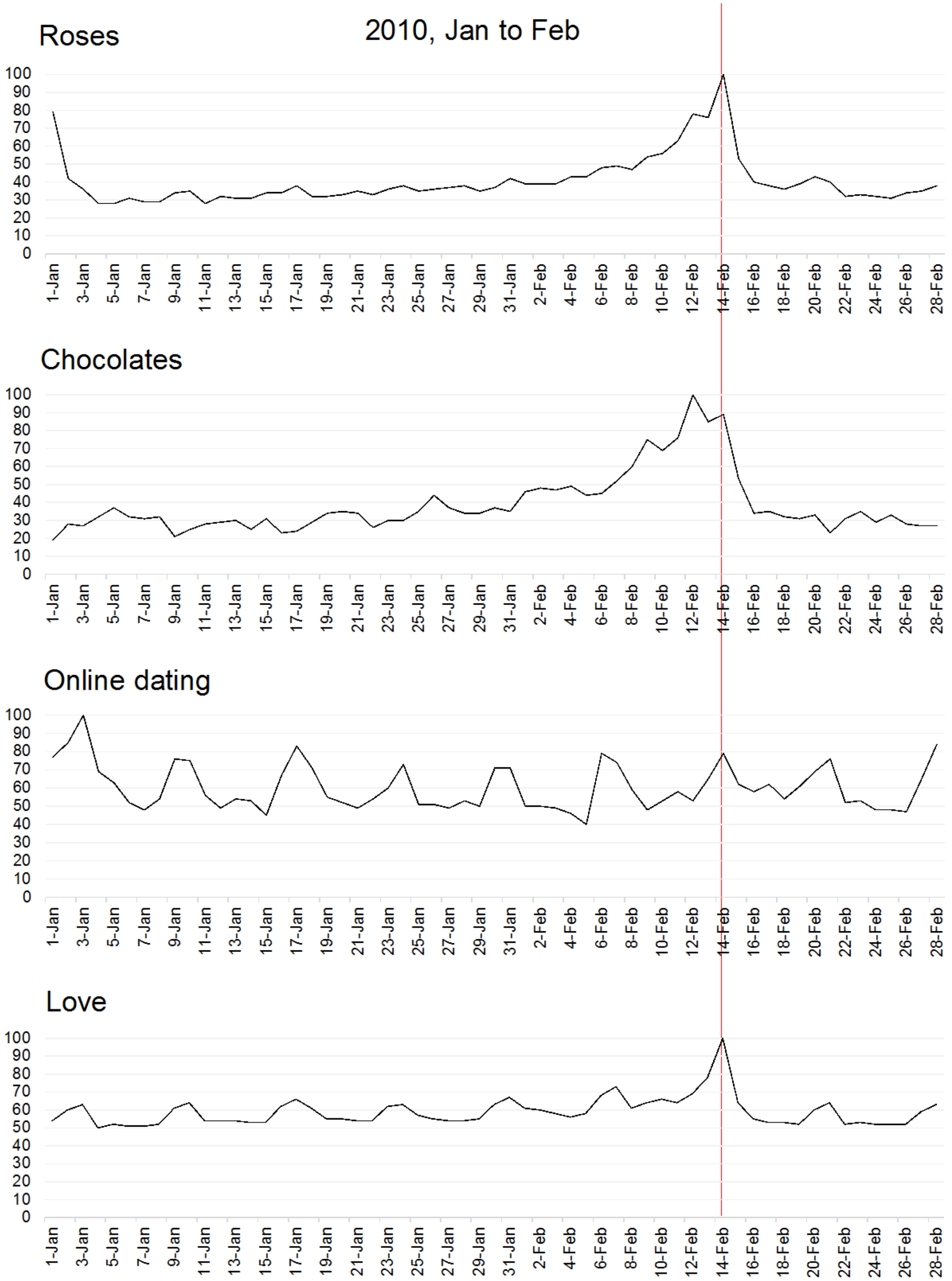

Figure S14. Population-level Google search term frequency for "roses" (panel a), "chocolates" (panel b), "online dating" (panel c), and "love" (panel d) between January and February, 2010. In each panel, the red vertical lines on the x-axis indicate Valentine's Day (February 14th) for the given year.

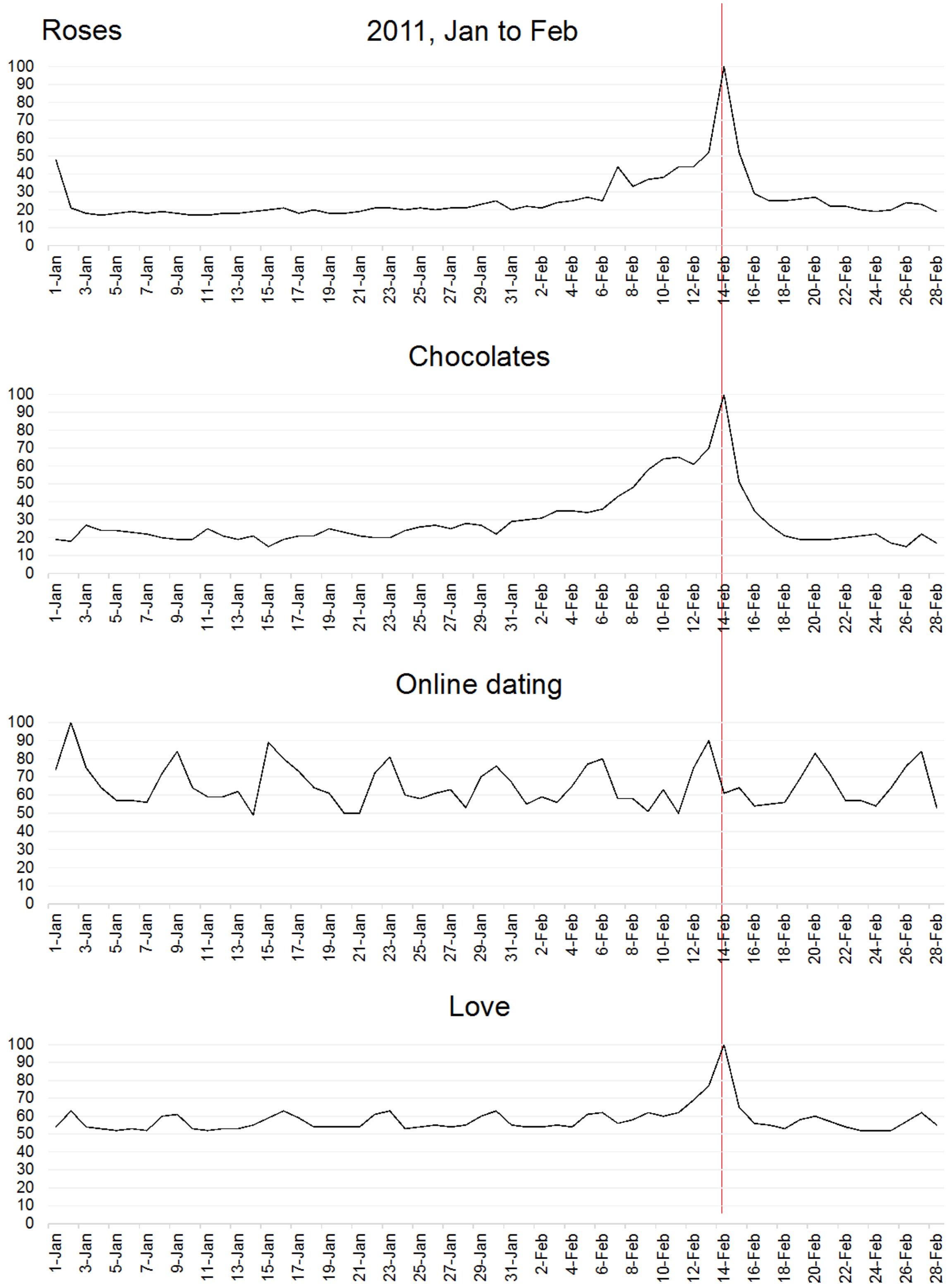

Figure S15. Population-level Google search term frequency for "roses" (panel a), "chocolates" (panel b), "online dating" (panel c), and "love" (panel d) between January and February, 2011. In each panel, the red vertical lines on the x-axis indicate Valentine's Day (February 14th) for the given year.

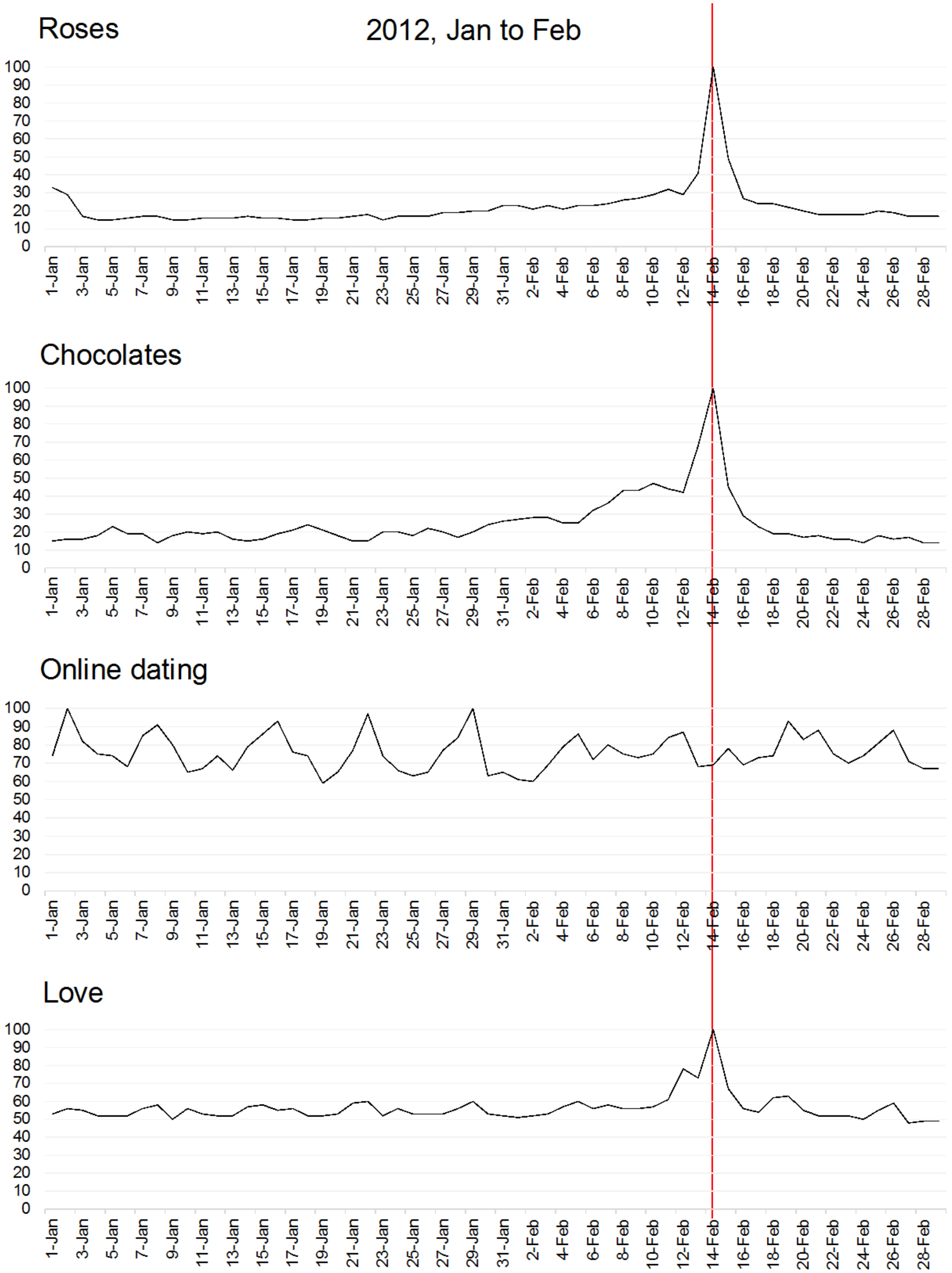

Figure S16. Population-level Google search term frequency for "roses" (panel a), "chocolates" (panel b), "online dating" (panel c), and "love" (panel d) between January and February, 2012. In each panel, the red vertical lines on the x-axis indicate Valentine's Day (February 14th) for the given year.

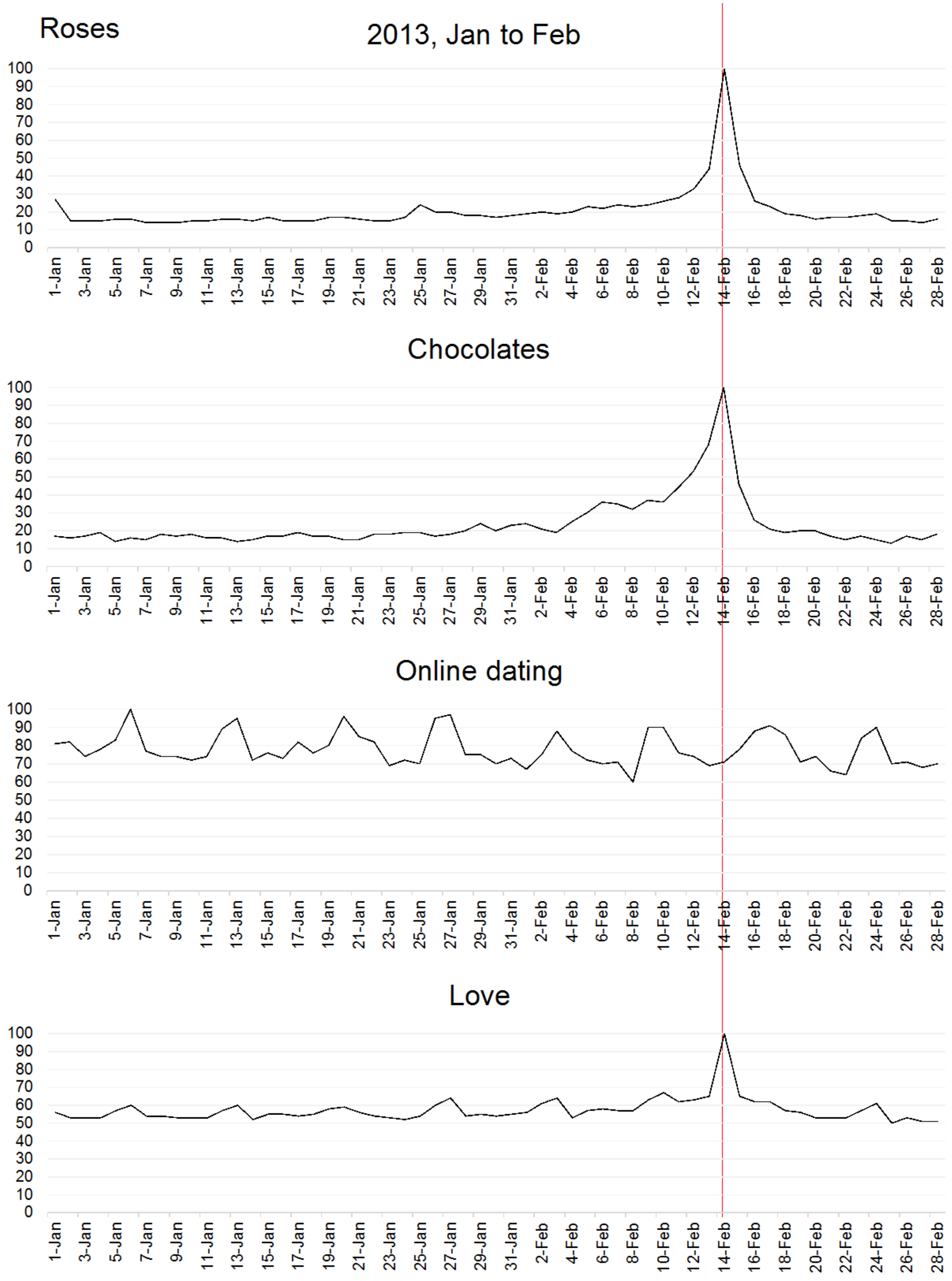

Figure S17. Population-level Google search term frequency for "roses" (panel a), "chocolates" (panel b), "online dating" (panel c), and "love" (panel d) between January and February, 2013. In each panel, the red vertical lines on the x-axis indicate Valentine's Day (February 14th) for the given year.

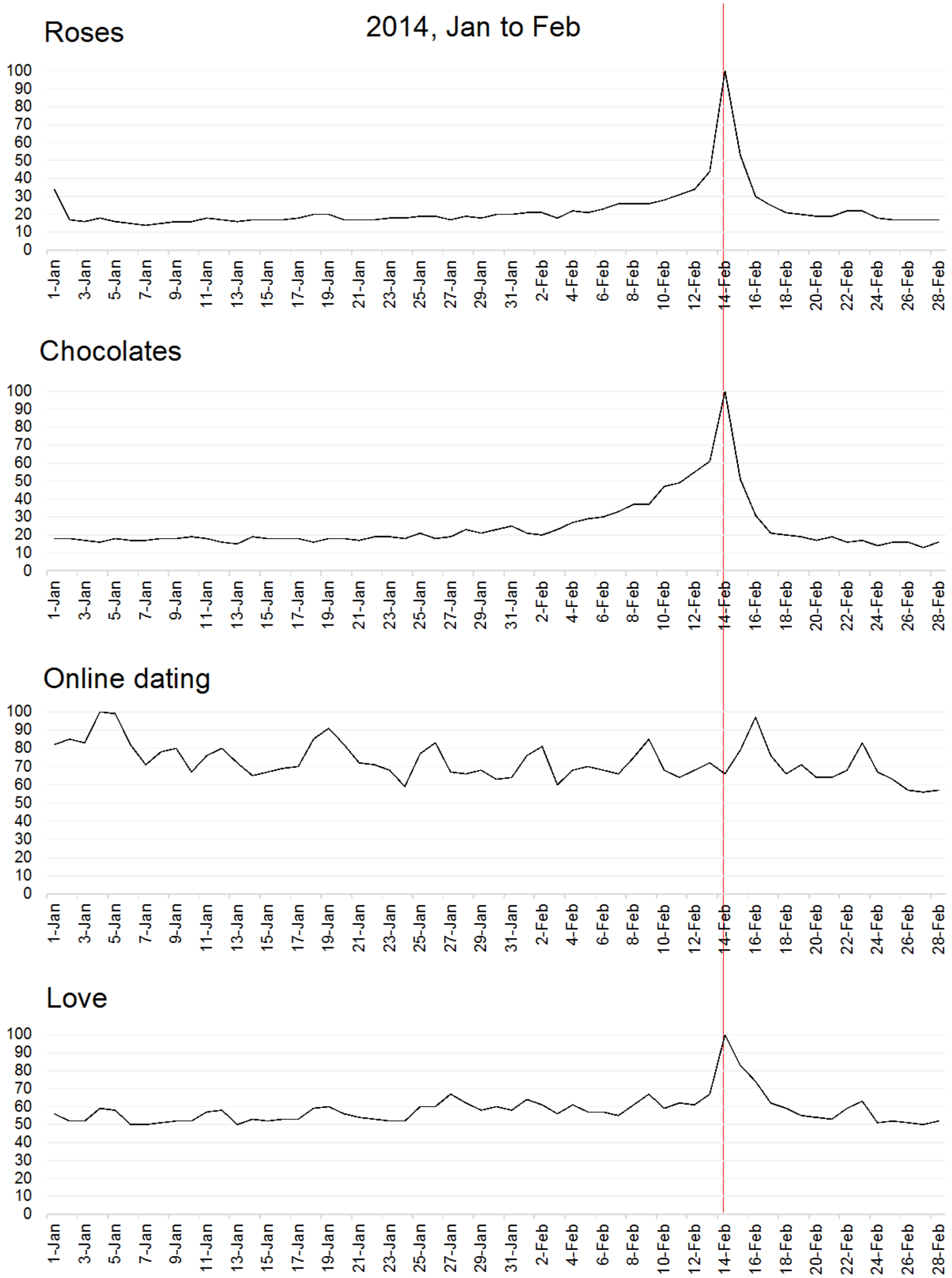

Figure S18. Population-level Google search term frequency for “roses” (panel a), “chocolates” (panel b), “online dating” (panel c), and “love” (panel d) between January and February, 2014. In each panel, the red vertical lines on the x-axis indicate Valentine’s Day (February 14th) for the given year.

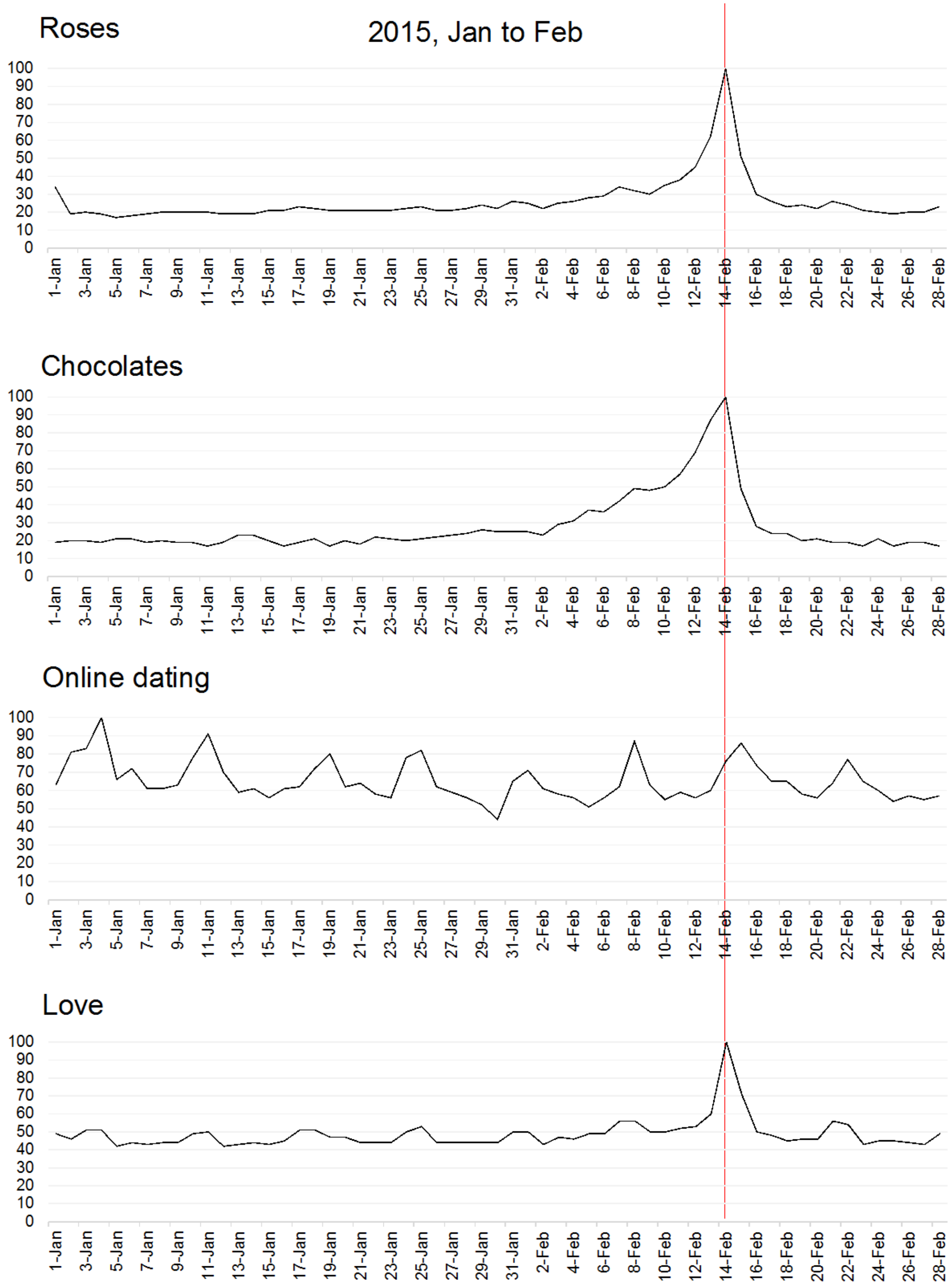

Figure S19. Population-level Google search term frequency for "roses" (panel a), "chocolates" (panel b), "online dating" (panel c), and "love" (panel d) between January and February, 2015. In each panel, the red vertical lines on the x-axis indicate Valentine's Day (February 14th) for the given year.

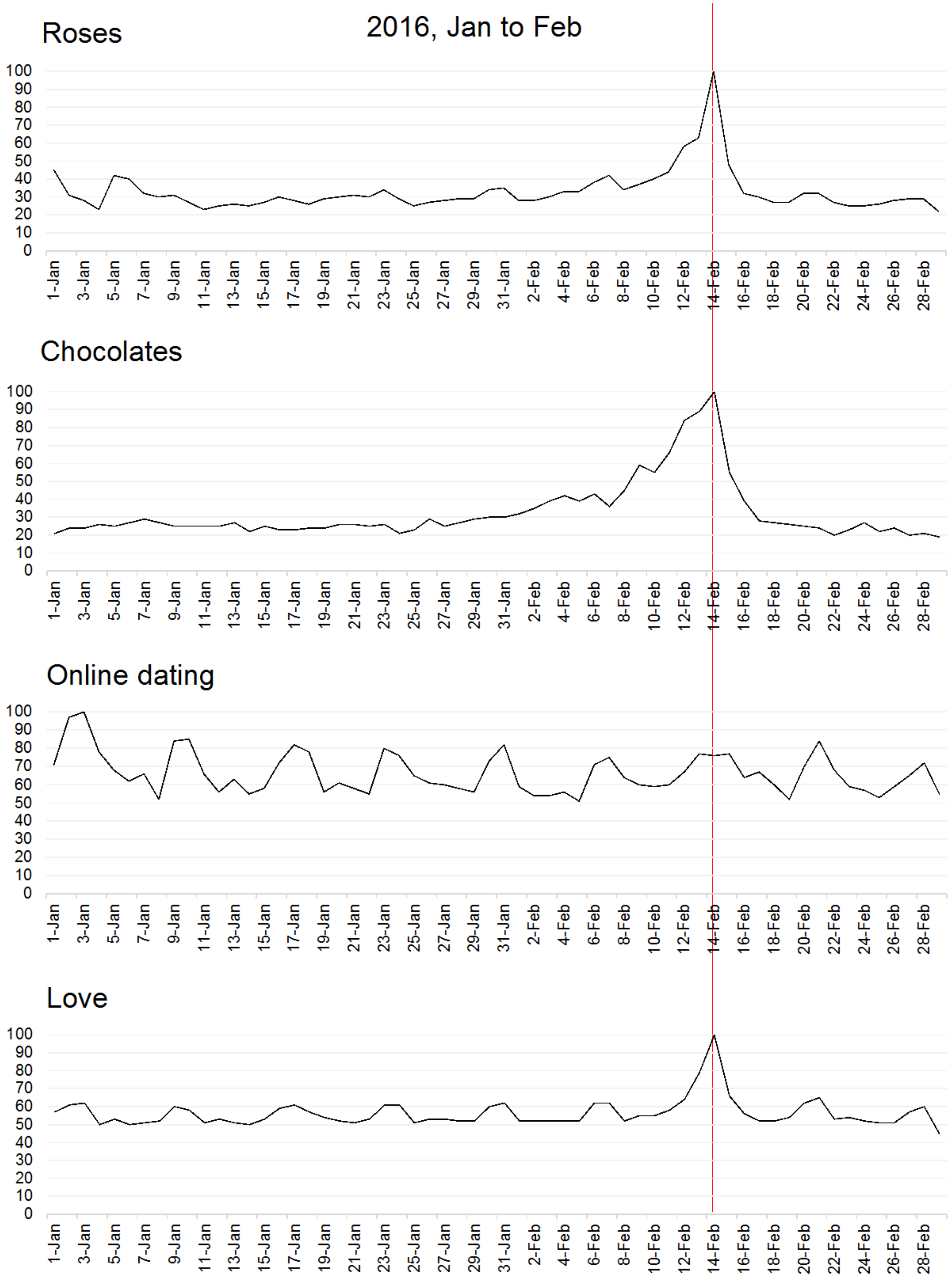

Figure S20. Population-level Google search term frequency for "roses" (panel a), "chocolates" (panel b), "online dating" (panel c), and "love" (panel d) between January and February, 2016. In each panel, the red vertical lines on the x-axis indicate Valentine's Day (February 14th) for the given year.

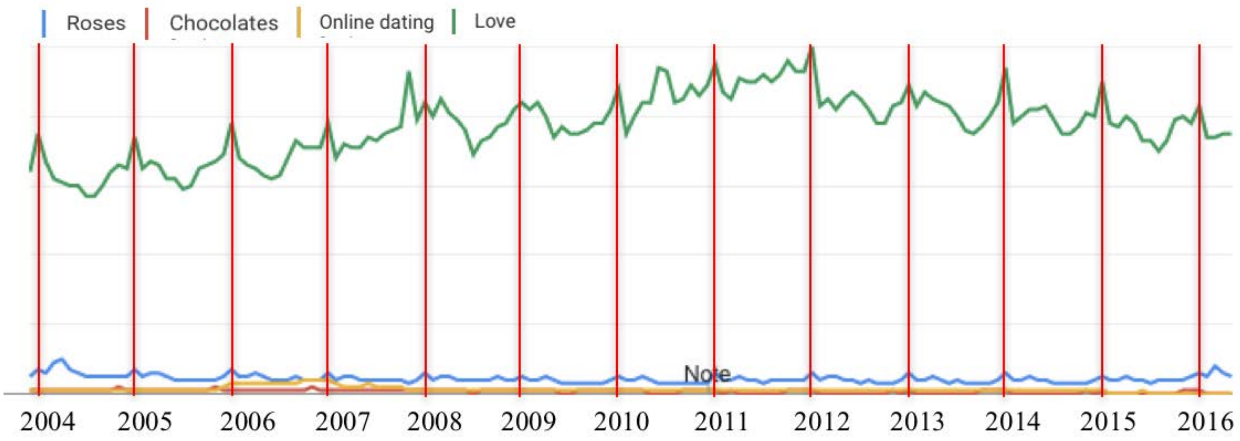

Figure S21. Population-level Google search term frequency between 2004-2016 for "love," "roses," "chocolates," and "online dating" when entered simultaneously in the Google Trends search query. The red vertical lines on the x-axis indicate Valentine's Day (February 14th) for the given year.

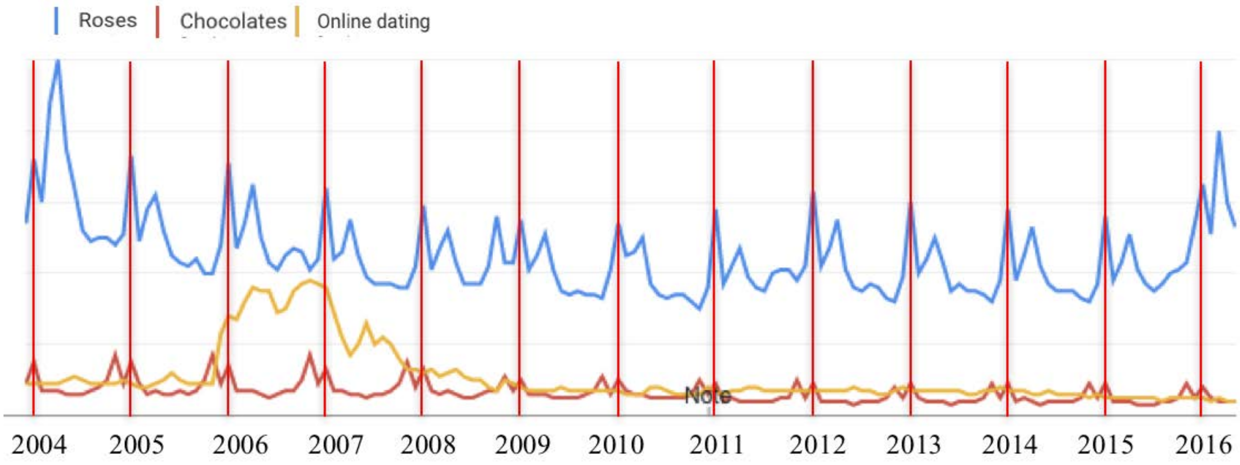

Figure S22. Population-level Google search term frequency between 2004-2016 for "roses," "chocolates," and "online dating" when entered simultaneously in the Google Trends search query. The red vertical lines on the x-axis indicate Valentine's Day (February 14th) for the given year.

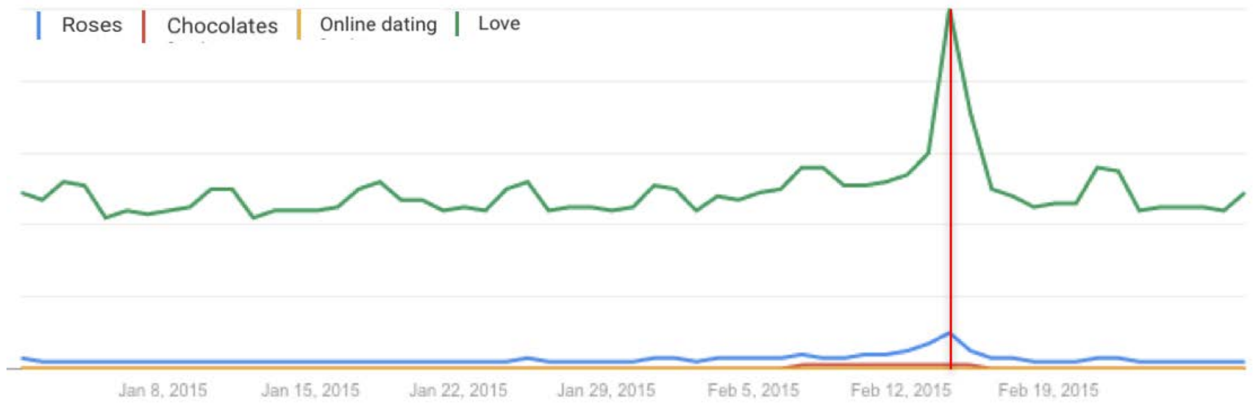

Figure S23. Population-level Google search term frequency between January and February 2015 for "love," "roses," "chocolates," and "online dating" when entered simultaneously in the Google Trends search query. The red vertical line on the x-axis indicates Valentine's Day (February 14th).

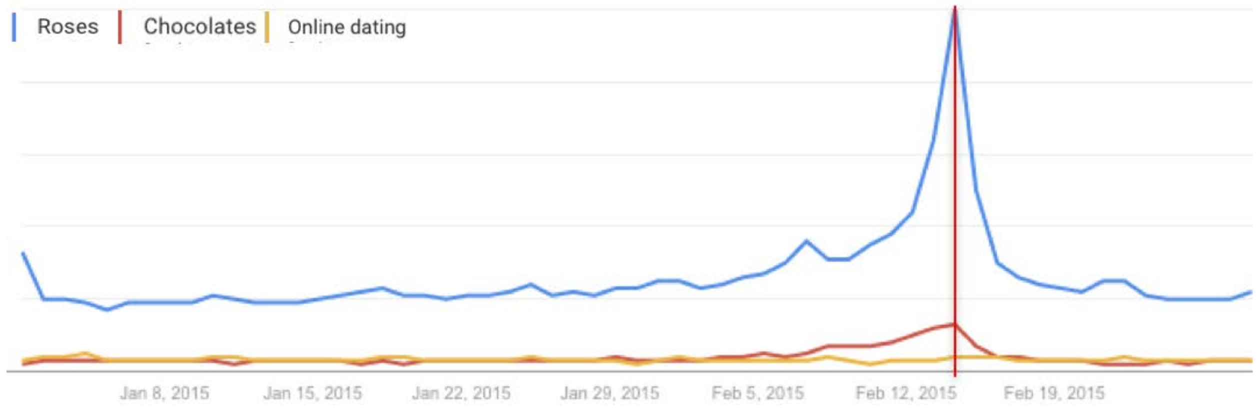

Figure S24. Population-level Google search term frequency for between January and February 2015 for "roses," "chocolates," and "online dating" when entered simultaneously in the Google Trends search query. The red vertical line on the x-axis indicates Valentine's Day (February 14th).
